# Systematic Study of the Surface Plasmon Resonance Signals Generated by Cells for Sensors with Different Characteristic Lengths

**DOI:** 10.1371/journal.pone.0107978

**Published:** 2014-10-23

**Authors:** Régis Méjard, Benjamin Thierry

**Affiliations:** Ian Wark Research Institute, University of South Australia, Mawson Lakes Campus, Mawson Lakes, South Australia, Australia; University of Zurich, Switzerland

## Abstract

The objectives of this study were to establish an in-depth understanding of the signals induced by mammalian cells in surface plasmon resonance (SPR) sensing. To this end, two plasmonic structures with different propagation and penetration distances were used: conventional surface plasmon resonance and long-range surface plasmon resonance. Long-range SPR showed a lesser sensitivity to the absolute number of round cells but a greater resolution due to its very narrow spectral dip. The effect of cell spreading was also investigated and the resonance angle of long-range SPR was mostly insensitive unlike in the conventional SPR counterpart. Experimental data was compared with suitable models used in the SPR literature. Although these simple averaging models could be used to describe some of the experimental data, important deviations were observed which could be related to the fact that they do not take into consideration critical parameters such as plasmon scattering losses, which is particularly crucial in the case of long-range SPR structures. The comparison between conventional and long-range SPR for cellular schemes revealed important fundamental differences in their responses to the presence of cells, opening new horizons for SPR-based cell assays. From this study, long-range SPR is expected to be more sensitive towards both the detection of intracellular events resulting from biological stimulation and the detection of microorganisms captured from complex biological samples.

## Introduction

Mainstream applications of surface plasmon resonance (SPR) biosensors typically involve the detection of (bio)molecules adsorbed, specifically or not, on the plasmonic sensing surfaces. SPR signals generated by the presence of biomolecule adlayers are, in first approximation, linearly related to the thicknesses, as long as the latter is uniform and much smaller than the evanescent probing fields. However, SPR sensing has also developed into a powerful technology for the sensing of large biological entities such as cells and bacteria. For instance, SPR sensing has been successfully used to detect the binding of pathogens and cells or monitor their responses to external triggers such as drug or signaling biomolecules (cell-based assay) [1], . SPR technology provides indeed a powerful means of studying the cellular response to stimulants. The signal generated in such experiments originates in complex biological events that locally impact on the refractive index distribution. Subsequent experiments involving upstream and downstream inhibitors of the stimulation or complementary techniques are therefore required to elucidate the biological meaning of the SPR response [7], [8].

An important consideration is that the presence of microorganisms on solid surfaces leads to layers inherently larger than the characteristic dimensions of plasmonic evanescent waves and consequently, resulting signals are not trivial. For instance, cells and bacteria extend well-beyond the probing fields in the *z* direction. The propagation length of the plasmons (propagation in the *x* direction, intersection of the plane of incidence and the sensor surface) is also expected to be a crucial parameter in regards to how the plasmonic waves actually sense the complex medium composed of cells and cover solution, as it was approached in the literature [9].

With respect to morphology-induced SPR signal, the data presently available in the literature is contradictory. While some studies have considered that cell-covered and cell-free regions of the sensors contribute to the overall signal independently [10], other studies make use of an effective refractive index for the cover medium composed of the solution and the cells [11]. The latter approach is the most commonly used one in waveguide biosensing [12], [13], [14]. Experimentally, splitting of SPR dips in presence of cells has been observed, and these *sub-dips* were associated to the co-existence of cell-free and cell-covered areas [9], [10]. These inconsistencies in both experimental and analytical reports are not surprising as to date, the effect of cells on the signal of SPR biosensors has not been systematically studied. A recent study also demonstrated that different parts of the SPR angular spectra reflect on different intracellular mechanisms (such as paracellular and transcellular) [15]. However, the aim of the present study is to systematically elucidate the structure-activity relationship of SPR sensors in presence of microorganisms and in the absence of external stimuli. We focussed more specifically on the relationship between the surface cellular density or morphology and the SPR response.

To this end, two different SPR structures were used in this work. The first one, conventional surface plasmon resonance (cSPR), is characterized by short propagation (and penetration) dimensions. The second one, long-range surface plasmon resonance (LRSPR), is characterized by long propagation and penetration dimensions. Since increases in cellular coverage can originate from either increases in the number of cells on the surface or from cellular spreading of a fixed number of cells, two systematic studies were designed to address these two different situations. The first involved round cells attached on the surfaces at different cell surface densities, which can be readily translated into cell coverage. In the following sections this scheme is referred to as *calibration*. The second situation considered the spreading of cells on the sensor surface and is referred to as *cellular spreading* scheme. Although, it has been reported that the spreading of cells was not a prominent feature in SPR signal [16], previous studies have used optical biosensing to evaluate spreading and determine cellular phase [11], suggesting the relevance of such biological events in SPR cellular schemes. In order to elucidate the effect of cellular spreading cells on plasmonic signals, cells were seeded at low density to minimize cell-cell interactions. Such interactions could, otherwise, mislead the signal interpretation. Low cell density is also expected to minimize the appearance of TM_0_ waveguide mode which would significantly increase the complexity of the system under study [11].

The second main objective of this study was to rigorously compare cell-induced signals for cSPR and LRSPR. This is of interest since LRSPR structures possess larger penetration depths, therefore the sensing electromagnetic (EM) fields can reach deeper into the cellular medium. Penetration depths for cSPR structures are of the order of 100–200 nm, whereas those of LRSPR are typically 500–1000 nm [17]. On the other hand, cSPR has better angular sensitivity than LRSPR with respect to bulk refractive index changes [18]. However, it has been recently reported that, in the case of bacterial detection, LRSPR is more sensitive than cSPR [19], [20]. To achieve a better understanding of the structure-activity relationship, a theoretical and experimental comparison of these two types of sensors is therefore provided in this study. Bridging this important knowledge gap will ultimately foster the application of SPR in the studies of microorganisms.

## Methods and Experimental

### Preparation of cSPR and LRSPR sensors

The cSPR sensors consisted of 1.5 nm of Cr and 50 nm of gold deposited in an HHV/Edwards TF600 sputter coater (Crawley, United Kingdom). LRSPR sensors consisted of 800 nm of spincoated fluoropolymer polydecafluoroxaheptadiene (Cytop) and 20 nm of gold. Cytop (CTL-809M, 9 wt %) and its solvent CT-SOLV 180 (perfluorotrialkylamine) were purchased from Asahi Glass (Tokyo, Japan). Both types of sensors were fabricated on N-LaSF9 glass substrates obtained from Hellma Optik (Jena, Germany). The sensors were sterilized by 5-min air plasma treatment followed by immersion in 70% Ethanol for 30 min.

### PDMS chambers for cell culture

Custom-made poly(dimethylsiloxane) (PDMS) chambers were used to culture cells on the SPR sensors. PDMS was obtained from Dow Corning (Midland, USA). The fabrication process relied on standard master-replica fabrication. Two wells per chamber were utilized: one, in which the cells were seeded, the other one was used as a control for the SPR measurements. The surface area of each well was 154 mm^2^. The height was approximately 4.5 mm. The PDMS chambers were sequentially cleaned with chloroform, acetone and water in a sonic bath to remove non-cross-linked, low-molecular weight, siloxane compounds which are toxic to cells [21]. The chambers were autoclaved prior to use.

### Preparation of devices for *calibration* experiments

The 3T3 fibroblast cell line was used as a model in this work and was purchased from ATCC. 3T3 are widely utilized in laboratory research, and were established from disaggregated tissue of an albino Swiss mouse embryo. They were selected for their ability to spread rapidly, as required in the second experimental scheme. The cells were cultured in complete media Dulbecco's modified eagle medium (DMEM) supplemented with glutamine and 10% (v/v) foetal bovine serum (FBS) at 37°C in a 100% humidified incubator with 5% CO_2_.

The sterilized SPR sensor chips were coated with poly-L-lysine (PLL) to enhance the cell-surface interactions. PLL was diluted to 0.5% (w/v) in phosphate buffer saline (PBS) (Sigma-Aldrich, St. Louis, USA). The PLL coating thickness was found to be approximately 2 nm on clean gold surfaces by SPR analysis. After mounting the PDMS chambers on the sensors, PLL solution was dispensed in the wells and left to adsorb for 30 minutes. The wells were then rinsed 3 times with PBS and then 3 times with the DMEM cell growth medium, and further incubated for an additional 60 minutes. Then the growth medium was replaced by PBS (3 rinsing steps). 3T3 cells were trypsinized and seeded into the custom-built wells at the desired concentrations calculated based on the targeted surface coverage. The cells were left to sediment on the sensors for 45 minutes at room temperature. The samples were then gently washed and fixed with a 4% formalin solution (Sigma-Aldrich) for 30 minutes. Both wells of the sensor devices were then gently washed 3 times with PBS. It can be noted that, from this procedure, the differences in adsorbed proteins and compounds on the control and cell surfaces were minimized in order not to bias the data interpretation.

### *Cellular spreading* experiments

The samples were prepared as described above until the cell seeding step. 3T3 cells suspended in phenol-free growth medium were left to sediment at room temperature for 45 minutes on the SPR sensor surfaces at a density of 150 cells/mm^2^. This corresponds to a surface coverage of 3%. The sample was then clamped into the SPR stage. The temperature was maintained at 37°C and the thermal stability was controlled by sequential measurements on the cell-free area of the sensor. After thermal equilibrium, the position was adjusted to place the cell-covered region in the beam's illumination. Spectra were acquired every 30 minutes to determine the effect of cell spreading on the SPR signal. The laser beam was blocked in between to minimize light exposure to the cells. The surface coverage was typically approximately 15% at the completion of the cell spreading. The experimental design and time-frame of the study (<8 hours) was chosen to take advantage of the absence of significant cellular proliferation immediately after reseeding. This ensured that only negligible variation in the cell number occurred during the experiment, which was verified experimentally. In parallel to the SPR study, automated time-lapse microscopy imaging of the spreading of the 3T3 cells on PLL treated glass was carried out and used as reference.

### Microscope and cell images

In order to calculate the density of cells on different samples, microscope images were taken and analysed either manually or with ImageJ software. The dimensions of the images were large enough to include many cells which enabled for reliable statistics. Typically at least 3 images were used and averaged. The cell sizes were calculated by measuring the number of pixels and using the pixel/micron conversion determined beforehand with a calibration grid. The density, initially expressed in number of cells/mm^2^ was then converted into coverage (%). The coverage was calculated as the projected surface area of an average cell multiplied by the number of cells in the image and divided by the surface area of the whole image. Lastly, from round 3T3 micrographs, the average diameter of a 3T3 cell was calculated to be 8.05 µm±1.5 µm (± represent the extrema). For the *cellular spreading* scheme, the cell projected surface areas were calculated by contouring the cells on each image of the time-lapse video using ImageJ. The contours of each cell in each image were manually drawn since the complex shapes that cells can make couldn't be dealt with by the software. Once the contours were drawn, the function measurement of ImageJ was used to determine the area within the contours. From these parameters the coverage could be calculated as described above. It was found, that when cells were spread on the surface, the average size of a spread cell was 722 µm^2^, corresponding to an effective diameter of 30.3 µm. Assuming a constant cell volume, this yielded averaged layers to be 2.3 µm±0.5 µm in thickness (in the *z* direction).

### SPR measurements and setup

**Figure 1:**
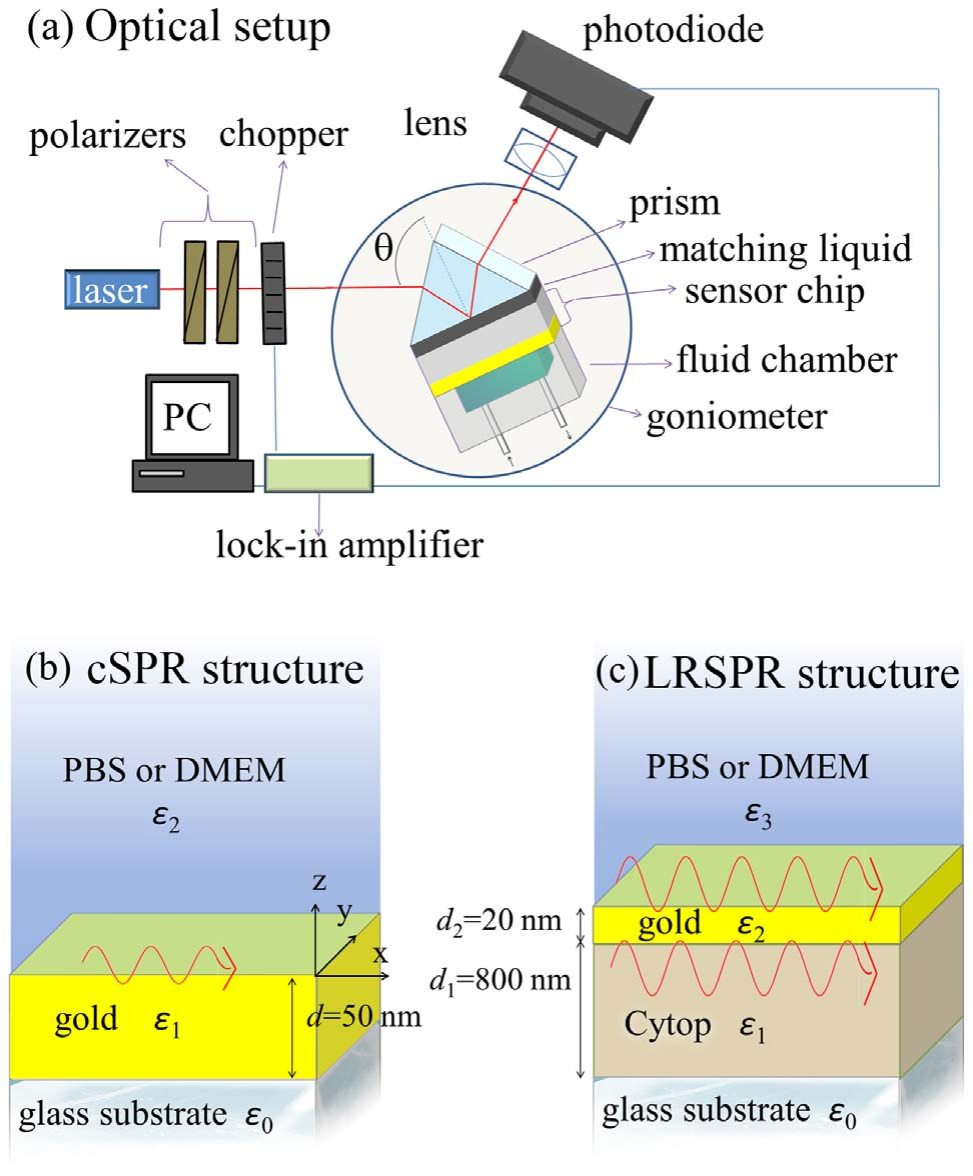
Experimental setup. (a) Scheme of the instrument set for angular interrogation of SPR sensors utilizing the attenuated total internal reflection method. Two different SPR structures with different characteristic propagation and penetration lengths were used in this study: (b) cSPR is composed of a layer of gold comprised between the glass substrate (prism) and the cover medium (PBS or DMEM plus cells) and (c) LRSPR is composed of a layer of Cytop polymer deposited on the glass substrate, onto which a thin layer of gold is sputtered, creating a RI symmetry supporting long range plasmon waves.

SPR measurements were conducted using an optical setup based on the attenuated total reflection (ATR) method with Kretschmann configuration that was developed at the Max Planck Institute for Polymer Research in Mainz (Germany). As shown in Figure 1a, a light beam at wavelength of *λ* = 632.8 nm from HeNe laser (CVI Melles Griot, USA) passed through two polarizers (Bernhard Halle Nachfl. GmbH, Germany) and a chopper (PerkinElmer Instruments, USA) before it was coupled to an LASFN9 glass prism. The intensity of the laser beam reflected from the prism base was detected by a photodiode detector and a lock-in amplifier (Signal Recovery, USA). The SPR sensor chip was optically matched to the prism base by using a refractive index matching oil (Cargille, USA) and the custom made PDMS flow-cell chamber was pressed against its surface. The angle of incidence, *θ*, of the laser beam hitting the SPR sensor surface was controlled by a motorized rotation stage (Hans Huber AG, Germany) and the whole system was controlled by the software WasPlas developed at the Max Planck Institute of Polymer Research (Germany).

The spectra were measured at controlled temperature of the sensing medium in a reproducible way. After SPR spectra were acquired, the parameters half width half maximum (*HWHM*), intensity at resonance (*R*_min_) and angle at resonance (*θ*_res_) were extracted from the curves using the program Winspall (Max Planck Institute, Mainz, Germany) or an algorithm written in Maple from Maplesoft (Waterloo, Canada). The *HWHM* were calculated on the left line of the SPR dip, that is the angles used in *HWHM* are *θ*_res_ and the angle for which the intensity is half the intensity span and which is lower than *θ*_res_. The values were taken for the dip of lower intensity in case two dips were present in the spectra. The *HWHM* was utilized for calculating the figure of merit as described in the first paragraph of the section Experimental results. To remove the bias introduced by minute fabrication differences between each sensor, the variation of these four parameters was used in this study. In the *calibration* scheme, the parameter values were subtracted by those for the PBS reference spectrum in the control well. In the *cellular spreading* scheme, the reference was the initial values obtained for non-spread cells (i.e. for coverage *c*≈3%).

In the following, the prism will be referred to as *medium 0* which is in contact to a LRSPR or cSPR structure, itself in contact to a cover solution. The cover solution is either 30°C PBS in the *calibration* experiments or 37°C DMEM in the *cellular spreading* ones (see Figure 1b and 1c).

The refractive indices of the different layers were: *n*_prism_ = 1.84662, *n*_cytop_ = 1.33675, *n*_PBS_(30°C) = 1.3384, *n*_DMEM_(37°C) = 1.3351.

## Experimental Results

### Comparison of cSPR and LRSPR in the *calibration* scheme

**Figure 2:**
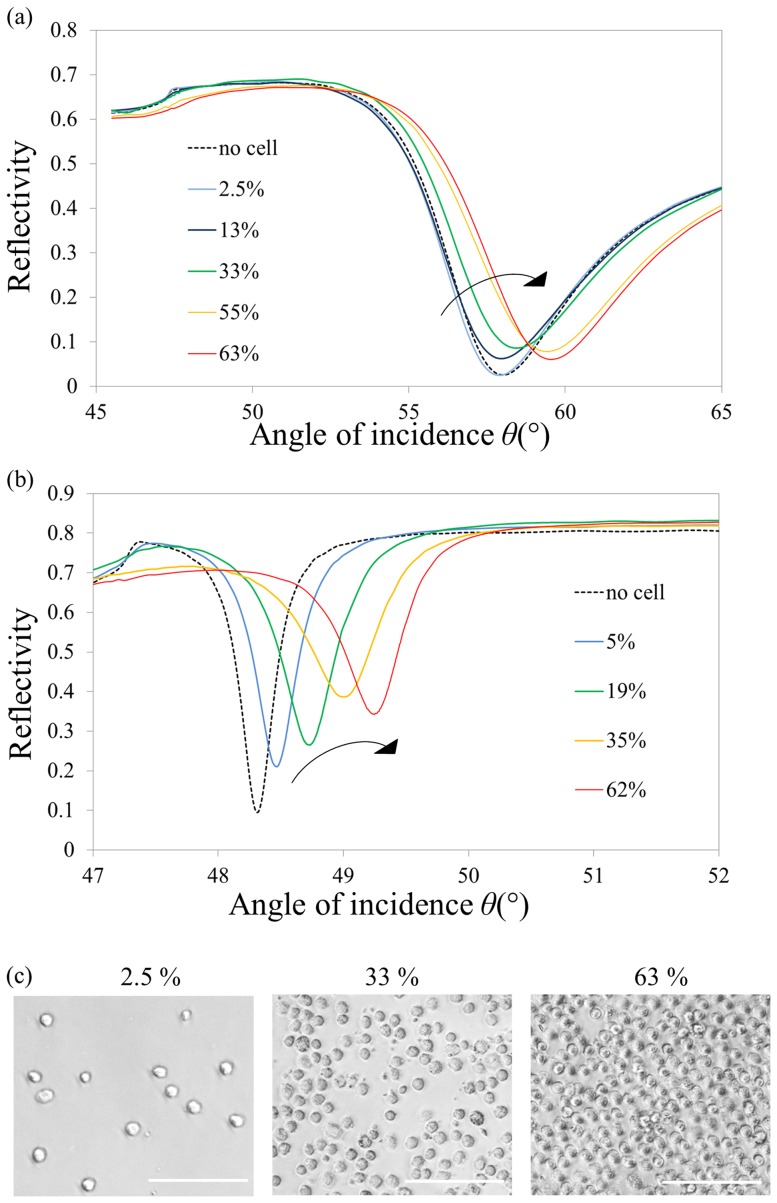
Experimental spectra recorded for cSPR sensors and LRSPR sensors at different cell coverage values. (a) cSPR spectra and (b) LRSPR spectra. The arrows indicate the evolution of the spectra. (c) Micrographs of three different cell coverage values of round 3T3 cells. Scale bar is 100 µm.

The experimental spectra obtained for both cSPR and LRSPR are presented Figure 2. A red shift was observed (resonance shifting towards higher angles) for both cSPR and LRSPR with increasing cell coverage. The intensity at resonance, *R*_min_, displayed a more complex evolution for both cSPR and LRSPR, with an initial increase at low densities before a decrease at higher cellular densities. A comparison of the spectrograms indicates a sharper distinction of these features for LRSPR spectra in comparison to cSPR ones.

**Figure 3:**
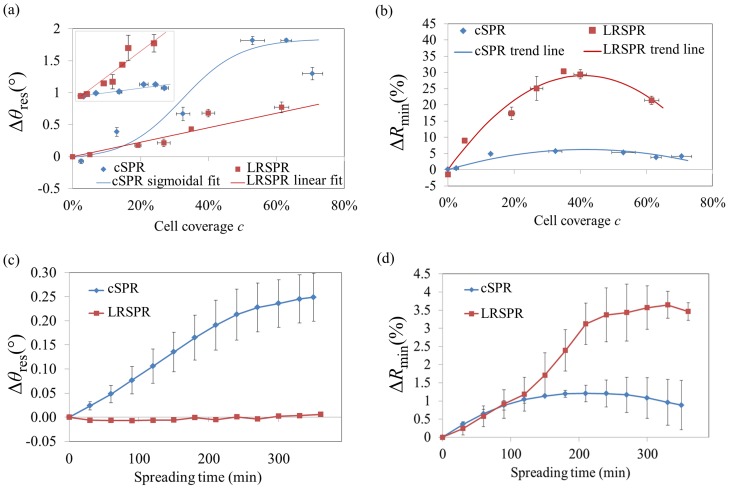
Comparison of cSPR and LRSPR spectra parameters for the two experimental schemes. Dependence of *θ*_res_ (a) and *R*_min_ (b) with respect to the cell coverage (round cells). The inset in (a) represents Δ*θ*_res_/*HWHM*, the slope of which is the figure of merit of the sensor. It was found that *FOM* = 1.30%^−1^ and *FOM* = 7.39%^−1^ for cSPR and LRSPR, respectively, yielding a 5.7-fold enhancement in the case of LRSPR. Dependence of Δ*θ*_res_ (c), and *R*_min_ (d) with respect to the cell spreading. The error bars represent the standard errors.

Figure 3a and 3b summarizes the evolution of the spectrum parameters, *θ*_res_ and *R*_min_, as a function of the cell coverage, *c*,(the other relevant parameters, half width at half maximum, *HWHM*, and the intensity at fixed angle *R*_fixed_, are plotted in Figure S1a and S1b). The angular sensitivity of cSPR to cellular coverage was found to be significantly higher than that of LRSPR. This finding is of interest since a recent study reported greater sensitivity of LRSPR for the detection of bacteria adsorbing on the sensors [19]. Our observation is, however, in agreement with the fact that, for homogeneous bulk changes, cSPR sensors present a sensitivity of ∼100°/RIU [18] whereas LRSPR sensors generally have a sensitivity of ∼25°/RIU [22]. However, since the *HWHM* is much smaller in the case of LRSPR, the localisation of *θ*_res_ can actually be more precise. When dealing with SPR sensitivity matters, it is therefore useful to consider the figure of merit (FOM), as it can be used as a tool to analyse the improvement in resolution of SPR-based sensors [22]. In this study the FOM is defined as *FOM* = *sens*_cell/_ *HWHM*, where *sens*_cell_ is the sensitivity of the sensors to the cell coverage on the surface. Although the full width at half maximum (FWHM) is often used, due to the asymmetry of SPR dips, using the *HWHM* is more precise and in turn can help minimizing potential discrepancies between FOM and the resolution calculated by refractometry taking into account the noise of blank signal [18].

The sensitivity to the cell coverage is defined in this study as the ratio: *sens*_cell_ = Δ*θ*_res_/Δ*c*, where Δ*θ*_res_ and Δ*c* correspond to angular shift and the increase in cellular coverage, respectively. Hence, *FOM* can be found directly by measuring the slope of Δ*θ*_res_/*HWHM* function of *c* (curves plotted in the inset of Figure 3a). Assuming linear relationships, it was found that *FOM*_cSPR_ = 1.30%^−1^ and *FOM*_LRSPR_ = 7.39%^−1^, which corresponds to a 5.7-fold improvement of LRSPR over cSPR sensors towards the detection resolution of microorganisms such as cells and bacteria, although one has also to take into consideration the experimental noise associated with SPR modalities to accurately predict their limits of detection.

### Comparison of cSPR and LRSPR in the *cellular spreading* scheme

**Figure 4:**
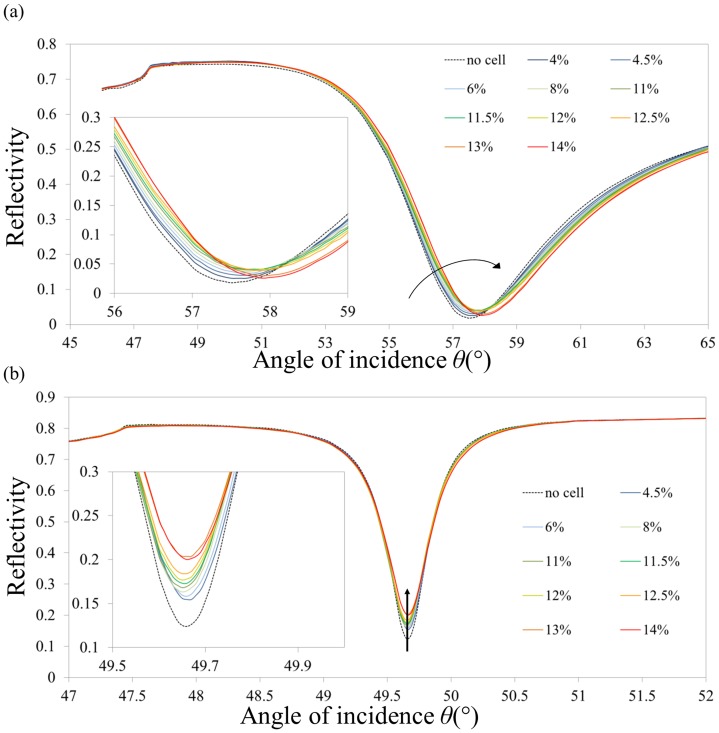
Typical reflectivity spectra of cSPR and LRSPR upon cellular spreading. (a) cSPR spectra and (b) LRSPR spectra. The cell seeding density was of 150 cells/mm^2^. The arrows show the evolution of the spectra. The insets show a close-up of the minimum of intensity. The coverage values were inferred from the curve in Figure S2.

Spectra for cSPR (a) and LRSPR (b) sensors obtained for various level of cell spreading are shown in Figure 4. Typical spreading time-lapse microscopic images can be found in Figure S2 as well as the associated trend of coverage versus spreading time, Figure S3. The coverage values determined by microscopy were used for the spectra of Figure 4.

Figure 3c and 3d summarizes the key spectrum parameters (Figure S1c and S1d show similar graphs for *HWMW* and *R*_fixed_). Interestingly, the angle of resonance, *θ*_res_, is sensitive to the spreading of cells only in case of cSPR. In the evolution of the *R*_min_ parameter, a similar trend is observed for cSPR and LRSPR at initial spreading but the cSPR *R*_min_ appears to saturate earlier than the *R*_min_ of LRSPR.

## Modelling and Comparison with Experimental Data

### Description of the models

To further understand and, in turn, predict the behaviours of the cSPR and LRSPR spectra in response to the presence of cells, theoretical curves from averaging models were compared to the experimental data.

Determining the refractive index (RI) of a cellular layer is a challenging endeavour, even though it has been done in some instances [23], [24]. Such value is inherently highly dependent on the method used from an optical standpoint (evanescent wave technique, refractometry, interferometry, etc.) as well as on the biological point of view (spreading of the cell, presence of cell-cell interactions, growth phase, passage number, etc.). The RI values used in the relevant literature to represent cellular layers are typically in the range of 1.35–1.37 RIU [24], [25], [26]. Therefore in the present study the values 1.35, 1.36 and 1.37 were used to model the presence of cells on the sensor surface in order to assess their impact on the SPR signal.

Two different methods of simulating SPR spectra were considered. First, the contributions of cell-covered and cell-free areas were assumed to be independent and their intensities were averaged as a function of the cell coverage, *c*. If *R* denotes the intensity of the normalized reflected p-polarized light, under the aforementioned assumption, one can write, following Ziblat *et al.* [10]:
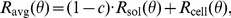
(1)where the subscript “avg” and “sol” stand for average and solution, respectively. This equation is only valid if the cell-covered and cell-free areas are independent with respect to the propagating surface plasmon waves. This means that the lateral dimension of the cells should be smaller than the characteristic propagation of the plasmons, i.e. the propagation length. According to Figure S4 and the discussion on propagation lengths in Appendix S1, it was found that this model should only be applied to cSPR structures.

In the second approach, an effective RI of the cover medium was assumed. It is comprised between that of the cover solution (e.g. PBS or DMEM) without cells, *n*_sol_, and that of a cover medium composed of a contiguous monolayer of cells, *n*_cell_. The condition to use the effective RI model is that the sensing waves feature long propagations compared to the cell lateral dimensions. Under this assumption, and based on the methodology first applied to waveguides, one can write [11], [14]:
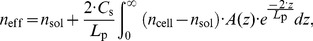
(2)where *A(z)* is the cross section of the cells at the distance *z* from the surface and *C*_s_ the number of cells per surface unit. *L*_p_ represents the penetration depth of the EM sensing fields into the medium (see paragraph S2 of Appendix S1). By using equation (2), one inherently assumes that the sensing EM fields penetrating into the cell-solution medium are evanescent, which is only true beyond the critical angle of the cellular medium, *θ*_c-cell_. In such a complex biological system, the critical angle is comprised between 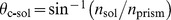, the critical angle of the solution, and 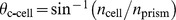, the critical angle of the cell, with *θ*_c-sol_<*θ*_c-cell_ and a significant part of the SPR dip may not necessarily be beyond *θ*_c-cell_ (especially in the case of narrow SPR dips such as those of LRSPR structures). Additionally, *L*_p_ varies substantially with the angle of incidence, *θ*. Hence, the expression of *n*_eff_ should strictly be a function of *θ* and should, moreover, be expressed by a different formula for angles smaller and greater than the critical angles.

The values for which *θ*<*θ*_c-sol_ are complex to model with an effective-RI and are not relevant since the parameters that characterize the SPR dips use values for angles beyond *θ*_c-sol_. Therefore the model will focus on values for angles greater than *θ*_c-sol_. The part of the spectrum for which *θ*_c-sol_≤*θ*<*θ*_c-cell_ can be determined by weighting *n*_cell_ and *n*_sol_ by the amount of EM intensity as a function of *z*:
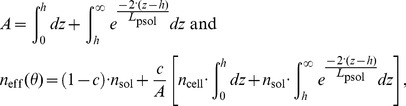
(3)where *h* is the average height of the cells, which are assumed to remain cylindrical (thus, 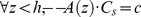). Eq. (3) can rewrite:
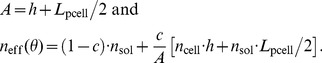
(4)When *θ*≥*θ*_c-cell_, one may use an equation featuring the weighting of *n*_cell_ and *n*_sol_ by the squared evanescent fields similar to Eq. (2). However, more rigorously, the penetration depths of each squared evanescent field – within the cell and beyond the cell – will be taken into account using *L*_psol_, the penetration depth in the solution, and *L*_pcell_, the penetration in the cell layer:
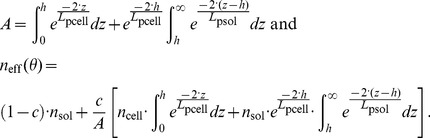
(5)
Equation (5) can rewrite:
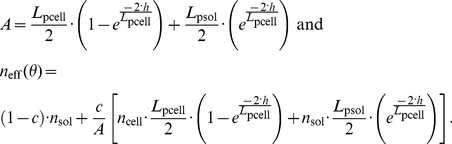
(6)According to the discussion on propagation lengths in Appendix S1, it was found that this model should only be applied to LRSPR structures.

### Comparison of the experimental and averaged-intensity modelled cSPR spectra

#### 1. *Calibration* scheme

**Figure 5:**
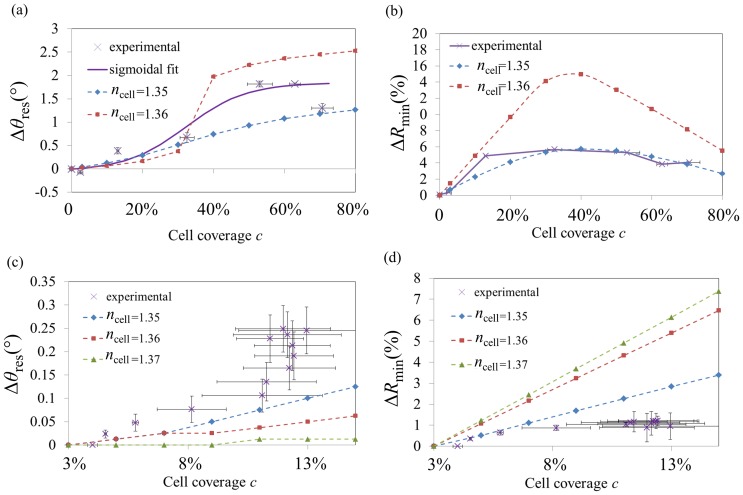
Comparison of cSPR parameters from experimental studies to those predicted by the averaged-intensity cSPR model. Dependence of *θ*_res_ (a) and *R*_min_ (b) with respect to the cell coverage. Dependence of *θ*_res_ (c), and *R*_min_ (d) with respect to the spreading of cells. The simulations are plotted in dashed curves (*n*_cell_ = 1.35: diamond, *n*_cell_ = 1.36: squares, *n*_cell_ = 1.37: triangles). Fitting the linear parts in (a) yielded *n*_cell_ = 1.3483 (by quadratic extrapolation of the slopes). Error bars represent the standard errors.

Using Eq. (1) for three different values of *n*_cell_ (*n*_cell_ = 1.35, *n*_cell_ = 1.36 and *n*_cell_ = 1.37), the simulation results show that by increasing the number of cells the characteristics of the spectra change significantly (see Figure S5). For all *n*_cell_, the increase of cell coverage leads to the angle of resonance (or apparent resonance), *θ*_res_, to shift to higher angles. In addition, in the case of *n*_cell_ = 1.37, the modelled cSPR spectra happened to split up into two sub-dips, one related to the uncovered areas (“PBS dip”) and one related to the cell-covered areas (“cell dip”). For *n*_cell_ = 1.36, the cSPR dips are getting misshaped as the coverage reaches ∼35%. The dip never splits up but shows a prominent feature (higher resonance coupling), on the PBS-dip side before 35% and on the cell-dip side after 35% coverage. For *n*_cell_ = 1.35, although no “dip-splitting” is predicted, a broadening of the dip is modelled as well as a rise of the intensity at resonance (*R*_min_), which both increase for low coverage values but then decrease for high coverage values. The resulting parameter analysis is presented in Figure 5a and 5b (Figure S6a and S6b show similar graphs for *HWMW* and *R*_fixed_). The data for the averaged-intensity model for n = 1.37 significantly deviated from the experimental data and were therefore not included in the figure for clarity. It can be noted that, similarly to homogenous adlayer formations on cSPR sensors, *θ*_res_ undergoes a red shift with increasing coverage. However, this shift doesn't follow a linear trend and can rather be fitted by a sigmoidal curve (in case of no dip splitting).

Comparing the experimental and theoretical parameters of the cSPR spectra in Figure 5a and 5b, reveals a good correlation with respect to the *R*_min_ parameters. Furthermore, the trend of Δ*θ*_res_ could be sigmoidal as shown by quality of the fitting (R^2^ = 0.976). Finally, from the experimental data of the Δ*θ*_res_, it can be concluded that the refractive index of the cells lies between *n*_cell_ = 1.35 and *n*_cell_ = 1.36.

#### 2. *Cellular spreading* scheme

Since the averaged-intensity model does not take into account the shape of cells, the spectra predicted for cellular spreading depends only on the cellular coverage and therefore follow the same trends as in Figure 5a and 5b but are limited to the coverage range from 3% to 15%. The parameter analysis of cSPR spectra is presented in the Figure 5c and 5d (Figure S6c and S6d show similar graphs for *HWMW* and *R*_fixed_). Quasi-linear behaviours are observed for most of the cases and any potential dip splitting has not occurred by the time the spread cells cover 15% of the surfaces. The comparison between the experimental data and the model data is not fully satisfactory.

### Comparison of effective-RI model with experimental LRSPR data

#### 1. *Calibration* scheme with LRSPR

LRSPR spectrum simulations under the effective-RI model are plotted in Figure S7. The behaviour of the SPR spectra is radically different from that that was simulated for the averaged-intensity model as the dip splits up in two for RI as low as *n*_cell_ = 1.35 (graphs can be found in Figure S8). In these simulations, LRSPR dips shift almost linearly. Furthermore no splitting and very little misshaping of the dips are predicted for all of the three *n*_cell_ values. In addition, *R*_min_ does not vary significantly.

**Figure 6:**
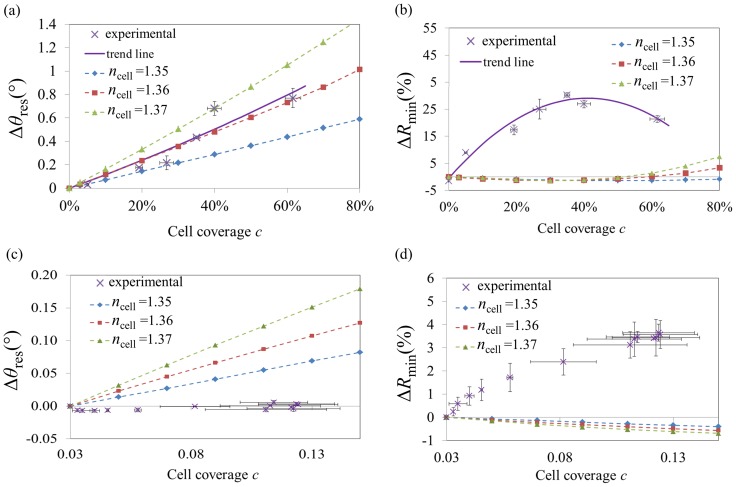
Comparison of LRSPR parameters from experimental studies to those predicted by the effective-RI LRSPR model. Dependence of *θ*_res_ (a) and *R*_min_ (b) with respect to the cell coverage. Dependence of *θ*_res_ (c) and *R*_min_ (d) with respect to the spreading of cells. The simulations are plotted in dashed curves (*n*_cell_ = 1.35: diamond, *n*_cell_ = 1.36: squares, *n*_cell_ = 1.37: triangles). The linear fitting in (a) yielded *n*_cell_ = 1.360. Error bars represent the standard errors.

The comparison of the experimental data with the effective-RI model is shown in Figure 6a and 6b (Figure S9a and S9b show similar graphs for *HWMW* and *R*_fixed_). The simulations showed a good agreement with the experimental angular shifts as the data could be fitted with a linear trend (R^2^ = 0.916). A poor correlation was, however, obtained for *R*_min_.

#### 2. *Cellular spreading* scheme with LRSPR

The comparison of the experimental data of LRSPR monitoring cellular spreading and the modelled data is presented Figure 6c and 6d (Figure S9c and S9d show similar graphs for *HWMW* and *R*_fixed_). It is worth noting that, according to the model, the *R*_min_ parameter varies only minimally in response to the spreading of cells, whereas *θ*_res_ does. Yet, the experimental data reveal a drastically different behaviour to what is predicted by the model.

## Discussion

### *Calibration* scheme

In the *calibration* scheme, which deals with increasing number of non-spread cells on the surface, one can observe an angular red shift, a convex behaviour for both *R*_min_ for both cSPR and LRSPR. However, according to this study the origin of these behaviours is expected to be different. The dip parameters of cSPR can be satisfyingly described with the averaged-intensity model for which one observes a sigmoidal variation of *θ*_res_, a convex variation of *R*_min_. This confirms the underlying hypothesis of Eq. (1) and implies that plasmonic waves propagating through the boundaries of cell-PBS interfaces contributes only little to the overall SPR signal which is in agreement with the fact that the propagation length is of similar dimension than the cell size. The observed discrepancies can be attributed to the fact that the propagation length of cSPR is not much smaller than the lateral dimension of round cells which put some stress on the assumption that cell-covered regions and cell-free regions contribute independently to the total reflectivity.

Furthermore, in previously reported publications, a SPR dip splitting was observed, which was not the case in our experimental data. Such dip splitting is predicted in the averaged-intensity model in the case of large RI contrasts between cells and cover solution. This situation can of course only happen for *L*_prop_ of the same order of magnitude or smaller than the lateral size of the cells. The presented effective-RI model does not support any SPR dip splitting.

Contrary to the cSPR, the variation of the spectrum parameters in LRSPR is expected to originate from an effective refractive index since the long-ranging plasmonic waves propagate much farther than the lateral size of the cells and therefore encounter many cell-free and cell-covered areas. In addition, this effective RI should be complex to take into account scattering losses due to the cell-PBS interfaces [15], however in this study the focus was brought on a real part of the effective RI similarly to what was found in the literature. The scattering losses are expected to account for the convex behaviour of *R*_min_ [11] but further in-depth studies are required to model these complex phenomena.

Nevertheless, assuming that scattering losses do not influence the position of *θ*_res_, the fitting of Δ*θ*_res_ yielded *n*_cell_ - *n*_sol_ = 0.022 in the case of LRSPR and *n*_cell_ - *n*_sol_≈0.017 for cSPR. The apparent higher RI measured by LRSPR can be explained by the fact that the EM fields of the LRSPR sensors probe deeper into the cell and can sense more of the high-RI intra cellular structures and membrane. Additionally, a lesser percentage of the evanescent tail of the LRSPR overlaps the nanometric gaps between the cell membrane and the substrate, typically considered to be in the 50 to 100 nm range [27]. The tendency of cSPR *HWHM* and *R*_min_ to have their maximum close to the 50% coverage is in agreement with the prediction of the averaged-intensity model as well as with previous reports including findings made for optical waveguides [28], [29]. Despite the theoretical challenges in understanding their complex behaviour, LRPSR sensors can be better tools to measure cellular concentration of round cell deposited on the surface as the narrowness of the dip translates into greater FOM over cSPR and the angular position is not affected by the spreading of cells.

The discrepancy between the present study's findings with mammalian cells, conveyed by Figure 3a, and those published in the literature with bacteria most likely do not originate from the difference in the bio-microobjects detected but rather their distance from the surface [19], [20]. Indeed LRSPR sensors have higher sensitivities towards the detection of material further away from the surface whereas cSPR perform best for material in contact to the surface. In this study, only minimalist surface chemistry was used to ensure cell adhesion to the gold surfaces. On the other hand, in the previously reported bacteria detection studies, complex surface chemistry could have contributed to displace the analyte further away from the surface with also a possibility of their being pushed away from the surface by their pili or flagella [30].

### *Cellular spreading* scheme

In this scheme, rather drastically different behaviours were observed for the two types of SPR structures. It is still reasonable to state that the averaged-intensity model can describe the spreading of cells in the case of cSPR. However noticeable discrepancies have appeared towards the end of the spreading phase. *θ*_res_ still shifts even though there is no more increase in cell size as observed in optical microscopy. On the one hand, this could be attributed to a greater sensitivity of SPR-based measurements, able to probe with a greater sensitivity cellular spreading than what can be observed in microscopy as suggested by studies relying on SPR imaging [27]. But this could also be attributed to the fact that cells are releasing microexudates at this stage without spreading further [12]. This situation could then be modelled by the deposition of a nanometric homogeneous adlayer, which would translate into angular shifts with small broadening of the dip (i.e. increase of *HWHM* – see Figure S6c) and no changes in *R*_min_, which corresponds to what is indeed monitored from 8% to ∼13% of cell coverage.

An interesting finding of this study is that *θ*_res_ is not sensitive to cellular spreading in the case of LRSPR. This could be explained by the fact LRSPR structures sense a proportion of cellular membrane (highest-RI component in the cell) that reduces significantly during the spreading of cells (due to the fact that LRSPR structures sense a large volume of the cells, including vertical edges of the cell). Hence, there might be more and more cellular material on the surface during the spreading, the LRSPR effectively considers this material with a lower and lower RI, and the two effects cancel out, leading to only minor fluctuations of *θ*_res_. Additionally, during cell spreading, the diffraction of the plasmons due to the cell-DMEM interfaces increases drastically because the cells do not spread in spherical caps (in fact the circularity drops down to ∼50% with 3T3 cells) which participates in the modifications of *R*_min_ while having little impact on *θ*_res_.

No inference of *n*_cell_ could be made for LRSPR. However, for cSPR, fitting the linear parts in Figure 5c yielded *n*_cell_≈1.348 (by quadratic extrapolation of the slopes). This means that *n*_cell_ - *n*_sol_≈0.013 for cells spreading on cSPR structures whereas *n*_cell_ - *n*_sol_≈0.017 for rounded cells. The apparent lower RI in the case of spreading cells most likely stems from the fact that the proportion of cell membrane over the cell volume is lower when cells are spreading.

## Conclusions

This study has been carried out in order to improve the understanding of the effects of cells on plasmonic signal in SPR biosensing and ultimately towards achieving a detailed structure-activity relationship. The first aim was to assess the validity of simple models that have been used previously in the literature for related optical sensing schemes. For SPR configuration when *L*_prop_ is of the same dimension as that of the cell lateral size (which is the case of cSPR sensors), the averaged-intensity model was found to provide a reasonably good prediction of SPR signals, taking in consideration some deviations in *R*_min_ parameter. On the other hand, when *L*_prop_ is much larger than the cell dimensions (LRSPR case) the model needed is based on the fact that an effective RI is sensed by the plasmonic waves. The effective-RI model could describe the angular shifting experimentally measured in the *calibration scheme* although it did not describe accurately the spreading of cells of the sensors. The difference between the two schemes was attributed to the importance of the cell membrane configuration and its apparent weight in the EM sensing fields, thereby bringing the focus on the issue of penetration depth in addition to the propagation length in the studied systems. However, even when the angular shifting was correctly described (*calibration* scheme), this model did not provided an accurate description of the parameters *R*_min_ of the spectra, which can be related to scattering loss in the system. Indeed, the main source of discrepancy between the data and the models is thought to originate from the scattering losses of the plasmonic waves encountering scatterers [11]. The implementation of scattering contribution in existing model is very challenging, especially since the vertical dimension of the scattering edge can vary from a few hundreds of nm for filopodia, to the whole cell's height. As a starting point for implementing plasmonic scattering into a model, one could consider making use of Rayleigh series' to describe the EM fields [31] or studying the propagation losses induced by refractive-index steps [32].

The second aim of the study was to compare cSPR and LRSPR in regards to their ability to “sense” the presence and morphological changes of cells on SPR sensors. It was observed that the coupling parameter *R*_min_ could be related to cellular coverage increase (associated to either cellular spreading or cell coverage increase), and is more sensitive in the case of LRSPR. Therefore, it can be concluded here that LRSPR can be used efficiently to monitor cellular spreading through the *R*_min_ parameter. Importantly, *θ*_res_ is not perturbed by the spreading of cells and therefore LRSPR sensors are expected to be more reliably used for the detection of intra-cellular signaling event without any unwanted influence of the spreading and other macroscopic morphological changes. It should also be possible in a single experiment to determine both the cellular surface concentration with no bias from cellular spreading, as well as to measure simultaneously cellular spreading by monitoring the parameter *R*_min_. This feature opens new horizons for cell-based assays. Finally, LRSPR offers a significantly better resolution with respect to the cell number (coverage of rounded cells) since the FOM is superior to that of cSPR by 5.7 fold. The latter together with the fact that the spreading of cells does not bias the measurement, justifies the use of LRSPR in biosensing approaches aimed at the detection of microorganisms from complex mixtures.

## Supporting Information

Figure S1 **Comparison of** ***HWHM*** **and** ***R*** **_fixed_ for cSPR and LRSPR in the two experimental schemes.** The parameter *R*_fixed_ represents the readable signal of typical biosensing experiments, it is obtained by the monitoring the intensity of the signal for a constant angle chosen to be close to, but smaller than, *θ*_res_, in the linear region of the SPR dip. Dependence of *HWHM* (a) and *R*_fixed_ (b) with respect to the cell coverage (round cells). Dependence of *HWHM* (c) and *R*_fixed_ (d) with respect to the cell spreading. The error bars represent the standard errors.
(TIF)

Figure S2 **Sequential images of the spreading of 3T3 cells.** (*t* = 0 min corresponds to seeding in the wells). From the analysis of the surface area covered by the cells, the trend of increase in cell coverage was inferred and plotted in Figure S3. Scale bar is 100 µm.
(TIF)

Figure S3 **Average cell coverage as a function of spreading time.** The complex behaviour of cellular spreading is linked to the fact that cells are constantly spreading, retracting and migrating on the surface. Nevertheless, up to about 3 hours after seeding, cells undergo an attachment and spreading phase. From 3 hours to approximately 7.5 hours the spreading slows down, possibly because of reaching its maximum. After 8 hours the coverage increases again and this can be attributed to proliferation of cells on the surface which is beyond the scope of this study. Error bars represent the standard errors.
(TIF)

Figure S4
**Plot of the propagation lengths of two LRSPR structures.** (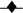) LRSPR composed of glass substrate, 850 nm of Cytop polymer, varying thickness of gold and PBS as the cover medium and (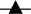) 800 nm of Cytop polymer, varying thickness of gold and PBS as the cover medium.(TIF)

Figure S5 **Simulated cSPR reflectivity intensities from the averaged-intensity model as function of cell coverage increase.** Different cell refractive indices were implemented: (a) ncell = 1.35, (b) ncell = 1.36 and (c) ncell = 1.37. The short-dashed curve represents the situation of the cover medium composed only by PBS and the long-dashed curve show the spectrum for a hypothetical monolayer of contiguous cells. A red shift occurs, however the angle of resonance shifts following a sigmoidal trend. Remarkably, the width parameter is described by a convex trajectory as well as *R*_min_. It can be noted that *R*_min_ recovers its initial value at the theoretical 100% coverage which is not the case for the width of the spectra. This is in agreement with the fact that the width of the SPR dip is function of the losses of the plasmonic structure and that any additional material on top of a SPR surface, with refractive index higher than that of the cover solution, will confine the EM fields more in the metal layer, which will increase the losses and broaden the SPR spectrum without changing the coupling efficiency, quantified by *R*_min_. (Raether H (1988) Surface plasmons on smooth surfaces. Surface Plasmons on Smooth and Rough Surfaces and on Gratings. Springer Berlin Heidelberg. pp. 4–39).
(TIF)

Figure S6 **Comparison of experimental cSPR** ***HWHM*** **and** ***R*** **_fixed_ to those predicted by the averaged-intensity cSPR model.** The parameter *R*_fixed_ represents the readable signal of typical biosensing experiments, it is obtained by the monitoring the intensity of the signal for a constant angle chosen to be close to, but smaller than, *θ*_res_, in the linear region of the SPR dip. Dependence of *HWHM* (a) and *R*_fixed_ (b) with respect to the cell coverage. Dependence of *HWHM* (c) and *R*_fixed_ (d) with respect to the spreading of cells. The simulations are plotted in dashed curves (*n*_cell_ = 1.35: diamond, *n*_cell_ = 1.36: squares, *n*_cell_ = 1.37: triangles). Fitting the linear parts in (a) yielded *n*_cell_ = 1.3483 (by quadratic extrapolation of the slopes). Error bars represent the standard errors.
(TIF)

Figure S7 **Simulated LRSPR reflectivity intensities from the effective-RI model (as per** **Eq. 4** **and** **6** **) as a function of cell coverage increase.** Different cell refractive indices were implemented: (a) ncell = 1.35, (b) ncell = 1.36 and (c) ncell = 1.37. The short-dashed curve represents the situation of the cover medium composed only by PBS and the long-dashed curve show the spectrum for a hypothetical monolayer of contiguous cells. The behaviours are close to those that one can found for bulk RI changes. The main contributor of the increase *R*_min_ is the RI mismatch between the cover medium and the polymer interlayer (Cytop) in the LRSPR structure. This translates by the coupling efficiency of the light into the plasmons departing from its optimum with increasing values of the cell coverage.
(TIF)

Figure S8 **Simulated LRSPR reflectivity intensities from the averaged-RI model (as per** **Eq. 1** **) as a function of cell coverage increase.** Different cell refractive indices were implemented: (a) *n*_cell_ = 1.35 and (b) *n*_cell_ = 1.37. The short-dashed curve represents the situation of the cover medium composed only by PBS and the long-dashed curve show the spectrum for a hypothetical monolayer of contiguous cells. A dip splitting is observed for *n*_cell_ as low as 1.35, which precludes any rational use of this model and confirms the hypothesis that LRSPR models should involve an effective refractive index.
(TIF)

Figure S9 **Comparison of experimental LRSPR** ***HWHM*** **and** ***R*** **_fixed_ to those predicted by the effective-RI LRSPR model.** The parameter *R*_fixed_ represents the readable signal of typical biosensing experiments, it is obtained by the monitoring the intensity of the signal for a constant angle chosen to be close to, but smaller than, *θ*_res_, in the linear region of the SPR dip. Dependence of *HWHM* (a) and *R*_fixed_ (b) with respect to the cell coverage. Dependence of *HWHM* (c) and *R*_fixed_ (d) with respect to the spreading of cells. The simulations are plotted in dashed curves (*n*_cell_ = 1.35: diamond, *n*_cell_ = 1.36: squares, *n*_cell_ = 1.37: triangles). The linear fitting in (a) yielded *n*_cell_ = 1.360. Error bars represent the standard errors.
(TIF)

Appendix S1 **Relationship between the plasmonic waves and the cells.**
(DOC)
