# Supplementary figures and images for: Systematic Study of the Surface Plasmon Resonance Signals Generated by Cells for Sensors with Different Characteristic Lengths
Source: PLoS One. 2014 Oct 23;9(10):e107978. doi: 10.1371/journal.pone.0107978 (PMC4207693; doi:10.1371/journal.pone.0107978)

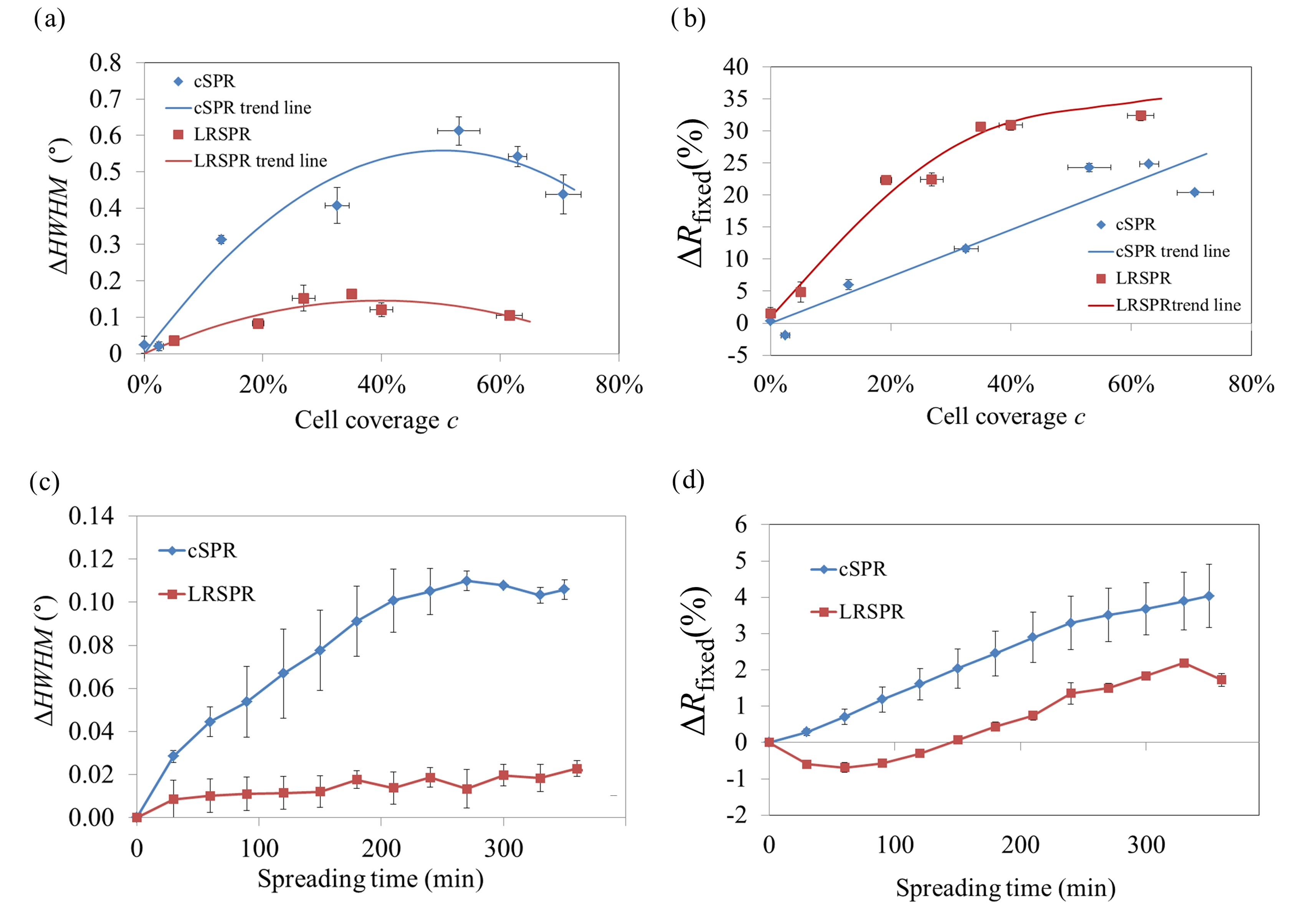

Supplement: Figure S1 — Comparison of HWHM and R fixed for cSPR and LRSPR in the two experimental schemes. The parameter R fixed represents the readable signal of typical biosensing experiments, it is obtained by the monitoring the intensity of the signal for a constant angle chosen to be close to, but smaller than, θ res, in the linear region of the SPR dip. Dependence of HWHM (a) and R fixed (b) with respect to the cell coverage (round cells). Dependence of HWHM (c) and R fixed (d) with respect to the cell spreading. The error bars represent the standard errors. (TIF) [file pone.0107978.s001.tif]

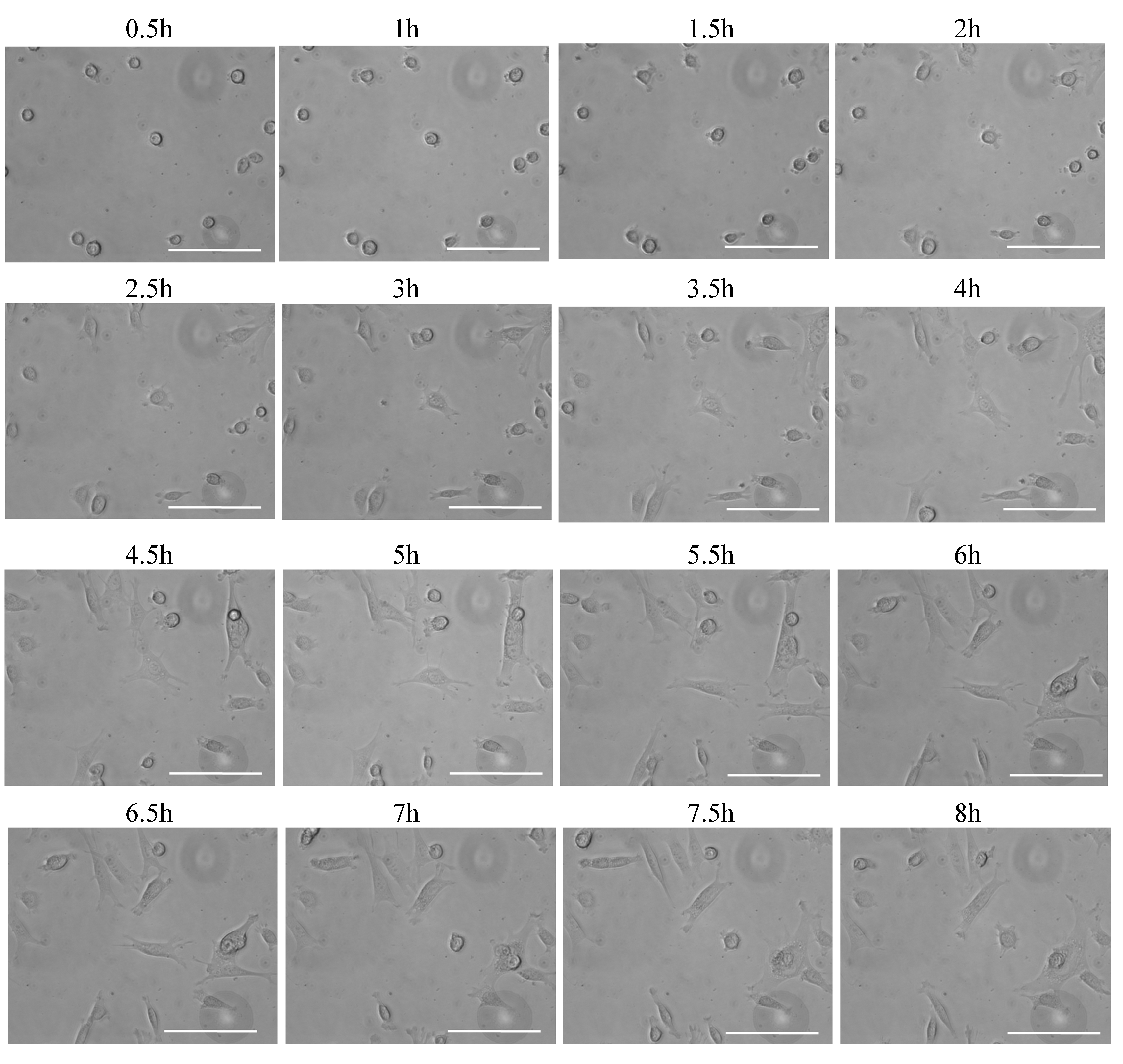

Supplement: Figure S2 — Sequential images of the spreading of 3T3 cells. (t = 0 min corresponds to seeding in the wells). From the analysis of the surface area covered by the cells, the trend of increase in cell coverage was inferred and plotted in Figure S3. Scale bar is 100 µm. (TIF) [file pone.0107978.s002.tif]

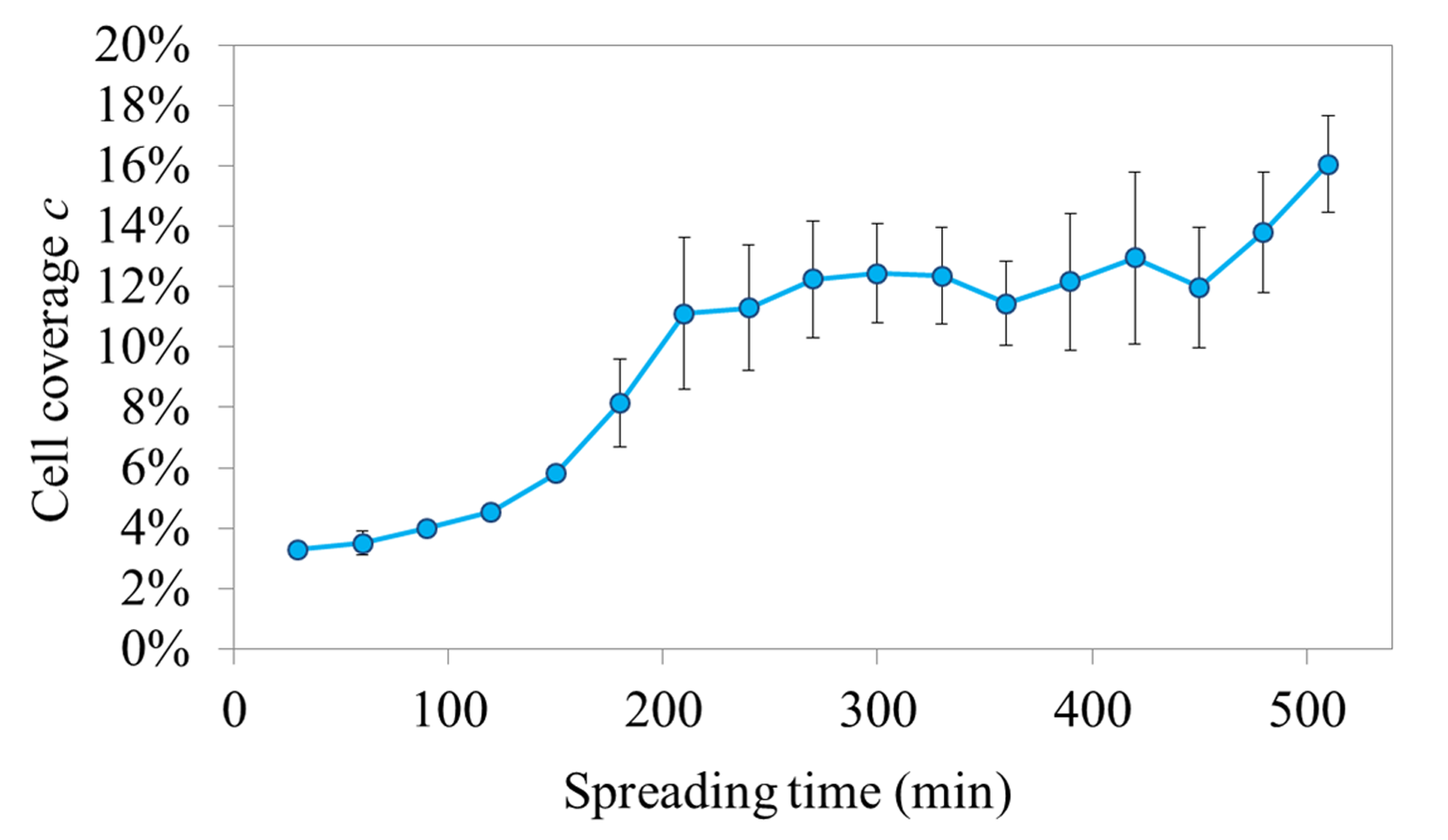

Supplement: Figure S3 — Average cell coverage as a function of spreading time. The complex behaviour of cellular spreading is linked to the fact that cells are constantly spreading, retracting and migrating on the surface. Nevertheless, up to about 3 hours after seeding, cells undergo an attachment and spreading phase. From 3 hours to approximately 7.5 hours the spreading slows down, possibly because of reaching its maximum. After 8 hours the coverage increases again and this can be attributed to proliferation of cells on the surface which is beyond the scope of this study. Error bars represent the standard errors. (TIF) [file pone.0107978.s003.tif]

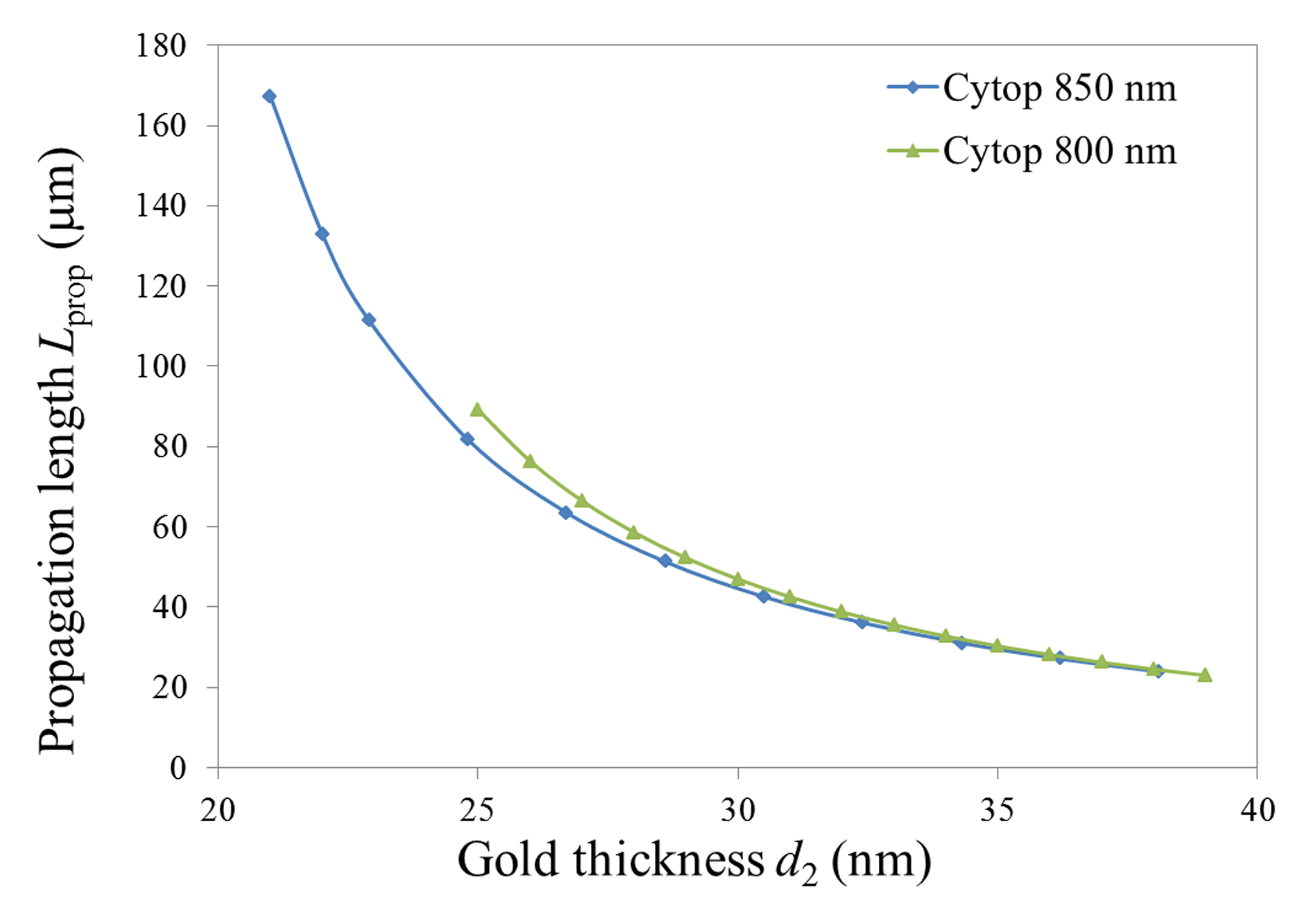

Supplement: Figure S4 — Plot of the propagation lengths of two LRSPR structures. () LRSPR composed of glass substrate, 850 nm of Cytop polymer, varying thickness of gold and PBS as the cover medium and () 800 nm of Cytop polymer, varying thickness of gold and PBS as the cover medium. (TIF) [file pone.0107978.s004.tif]

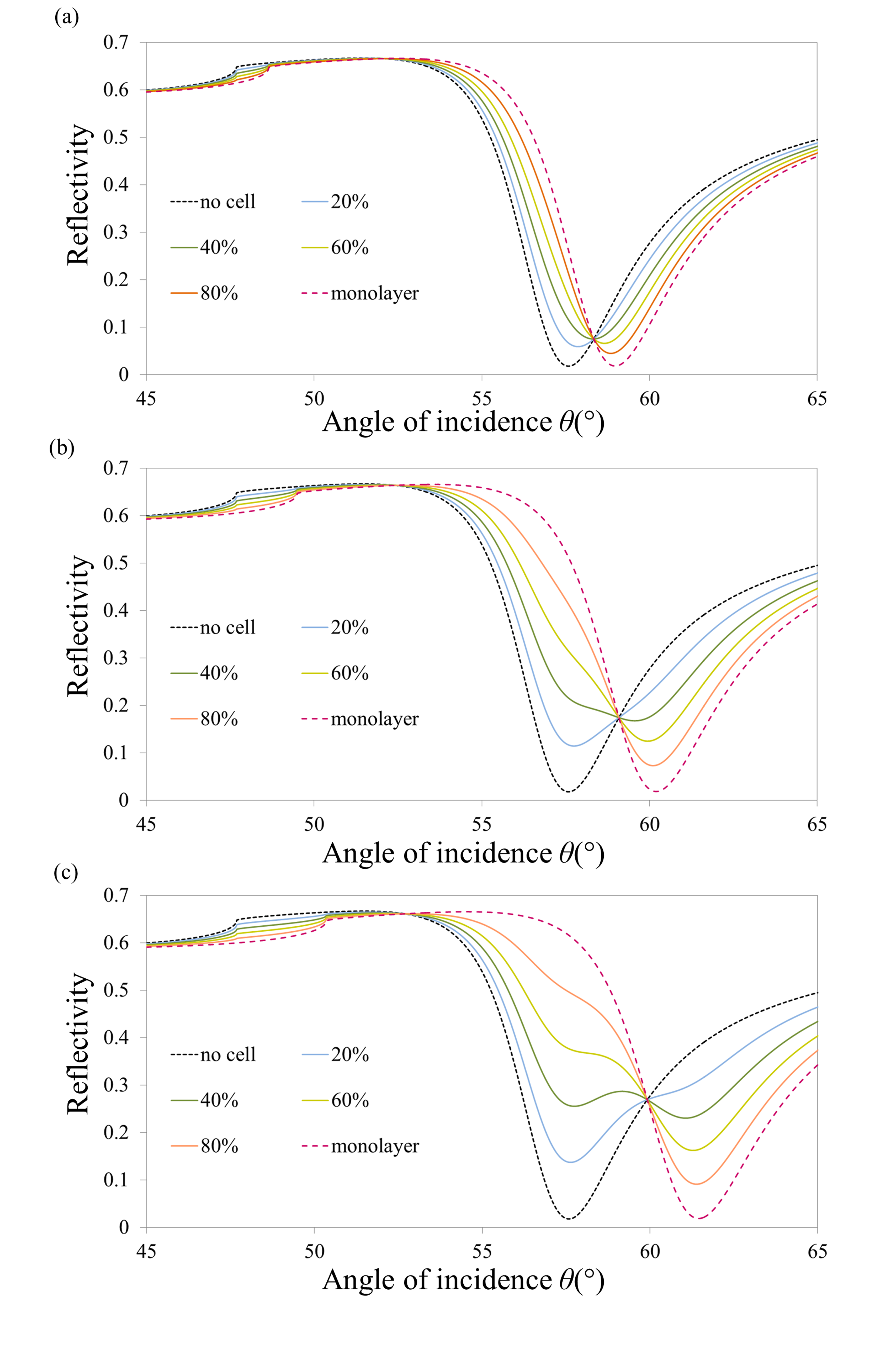

Supplement: Figure S5 — Simulated cSPR reflectivity intensities from the averaged-intensity model as function of cell coverage increase. Different cell refractive indices were implemented: (a) ncell = 1.35, (b) ncell = 1.36 and (c) ncell = 1.37. The short-dashed curve represents the situation of the cover medium composed only by PBS and the long-dashed curve show the spectrum for a hypothetical monolayer of contiguous cells. A red shift occurs, however the angle of resonance shifts following a sigmoidal trend. Remarkably, the width parameter is described by a convex trajectory as well as R min. It can be noted that R min recovers its initial value at the theoretical 100% coverage which is not the case for the width of the spectra. This is in agreement with the fact that the width of the SPR dip is function of the losses of the plasmonic structure and that any additional material on top of a SPR surface, with refractive index higher than that of the cover solution, will confine the EM fields more in the metal layer, which will increase the losses and broaden the SPR spectrum without changing the coupling efficiency, quantified by R min. (Raether H (1988) Surface plasmons on smooth surfaces. Surface Plasmons on Smooth and Rough Surfaces and on Gratings. Springer Berlin Heidelberg. pp. 4–39). (TIF) [file pone.0107978.s005.tif]

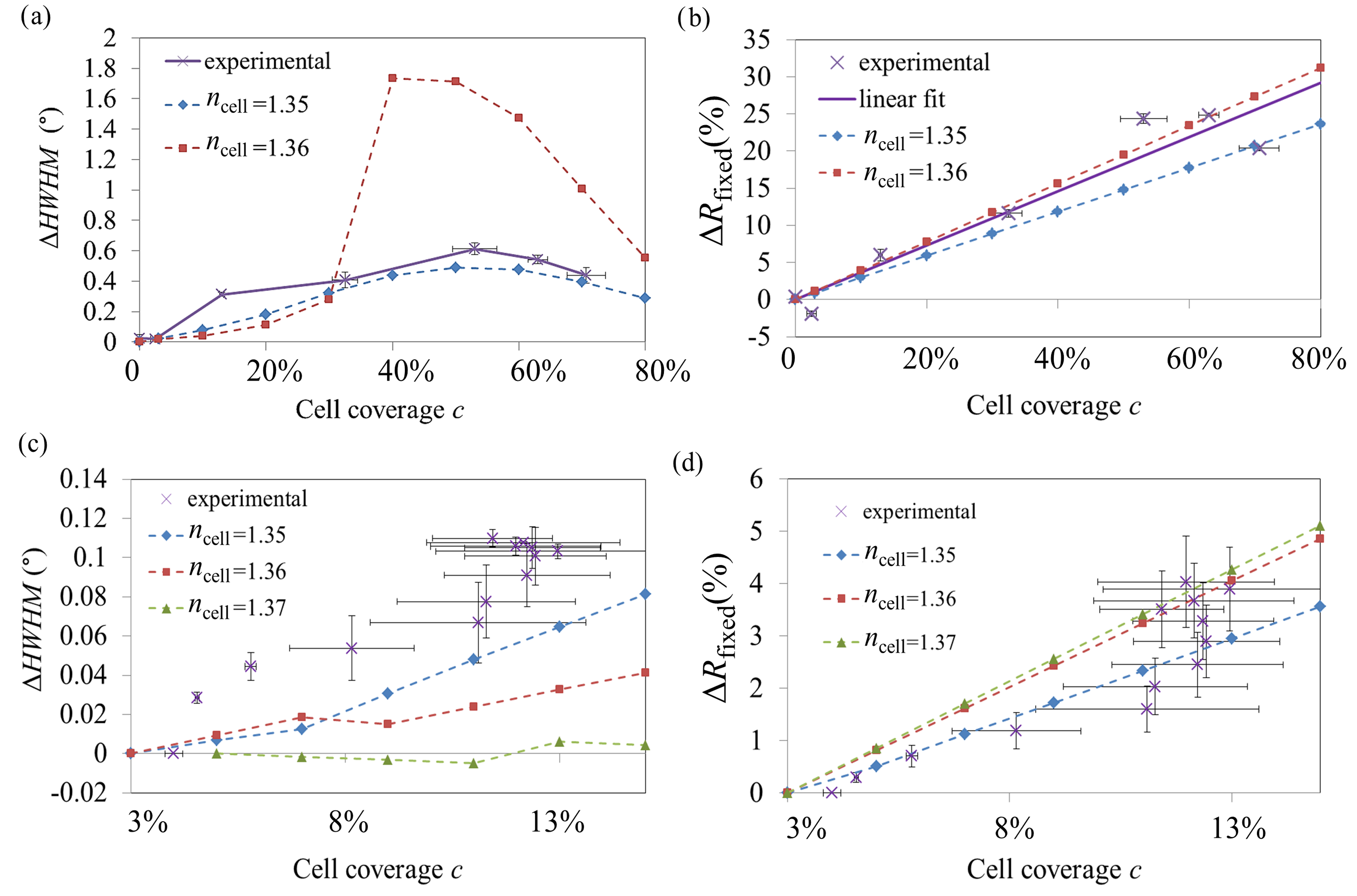

Supplement: Figure S6 — Comparison of experimental cSPR HWHM and R fixed to those predicted by the averaged-intensity cSPR model. The parameter R fixed represents the readable signal of typical biosensing experiments, it is obtained by the monitoring the intensity of the signal for a constant angle chosen to be close to, but smaller than, θ res, in the linear region of the SPR dip. Dependence of HWHM (a) and R fixed (b) with respect to the cell coverage. Dependence of HWHM (c) and R fixed (d) with respect to the spreading of cells. The simulations are plotted in dashed curves (n cell = 1.35: diamond, n cell = 1.36: squares, n cell = 1.37: triangles). Fitting the linear parts in (a) yielded n cell = 1.3483 (by quadratic extrapolation of the slopes). Error bars represent the standard errors. (TIF) [file pone.0107978.s006.tif]

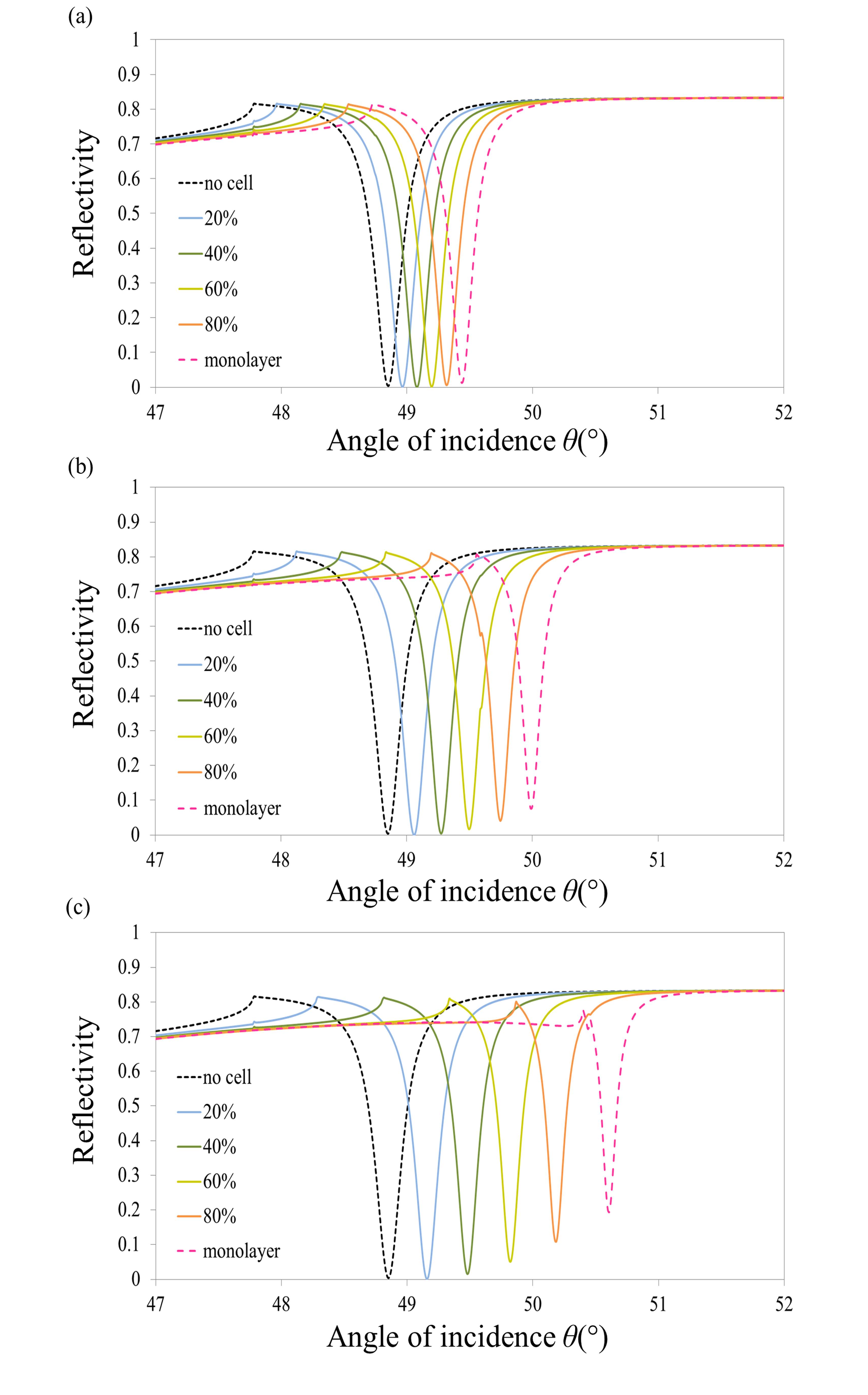

Supplement: Figure S7 — Simulated LRSPR reflectivity intensities from the effective-RI model (as per Eq. 4 and 6 ) as a function of cell coverage increase. Different cell refractive indices were implemented: (a) ncell = 1.35, (b) ncell = 1.36 and (c) ncell = 1.37. The short-dashed curve represents the situation of the cover medium composed only by PBS and the long-dashed curve show the spectrum for a hypothetical monolayer of contiguous cells. The behaviours are close to those that one can found for bulk RI changes. The main contributor of the increase R min is the RI mismatch between the cover medium and the polymer interlayer (Cytop) in the LRSPR structure. This translates by the coupling efficiency of the light into the plasmons departing from its optimum with increasing values of the cell coverage. (TIF) [file pone.0107978.s007.tif]

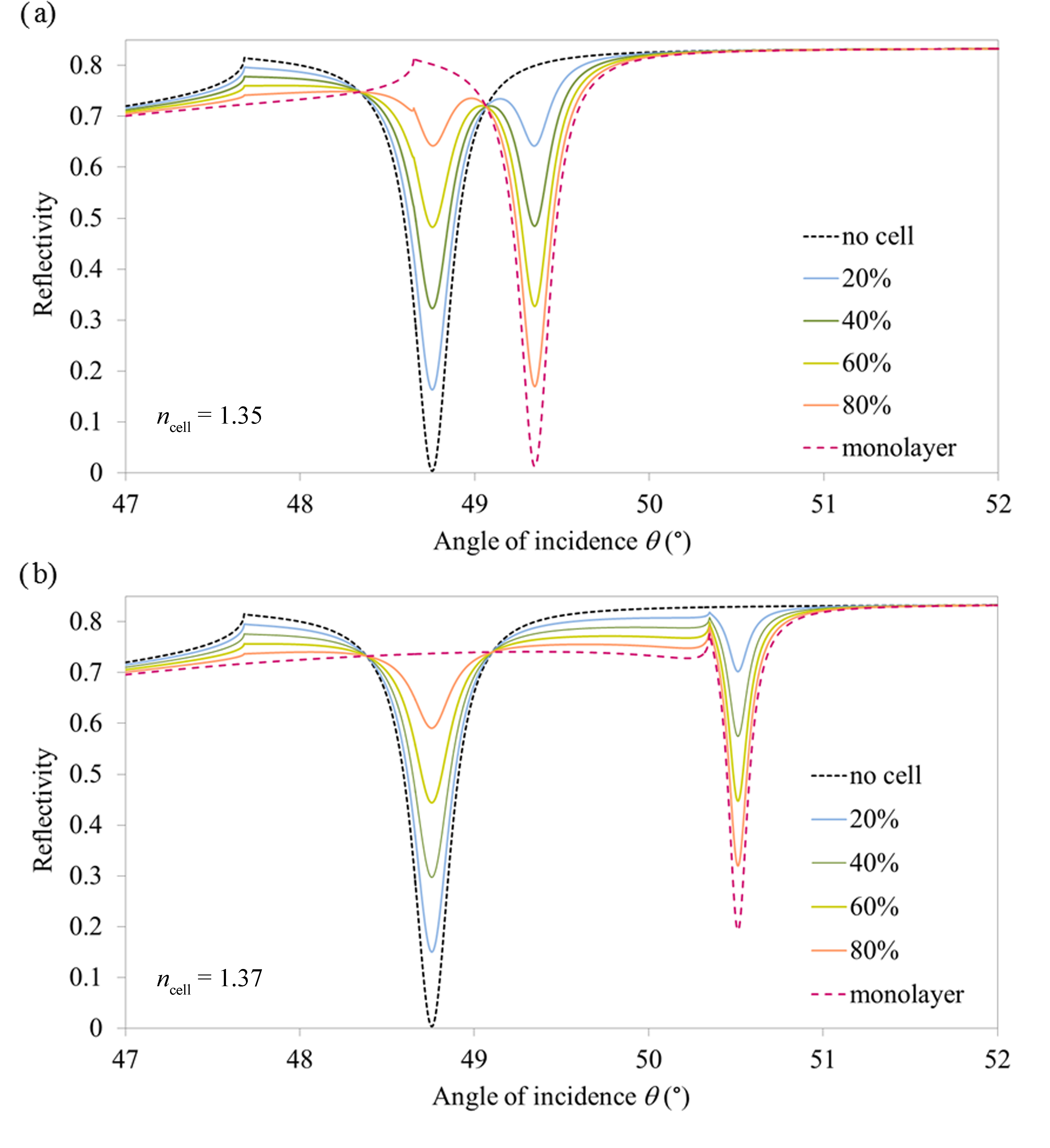

Supplement: Figure S8 — Simulated LRSPR reflectivity intensities from the averaged-RI model (as per Eq. 1 ) as a function of cell coverage increase. Different cell refractive indices were implemented: (a) n cell = 1.35 and (b) n cell = 1.37. The short-dashed curve represents the situation of the cover medium composed only by PBS and the long-dashed curve show the spectrum for a hypothetical monolayer of contiguous cells. A dip splitting is observed for n cell as low as 1.35, which precludes any rational use of this model and confirms the hypothesis that LRSPR models should involve an effective refractive index. (TIF) [file pone.0107978.s008.tif]

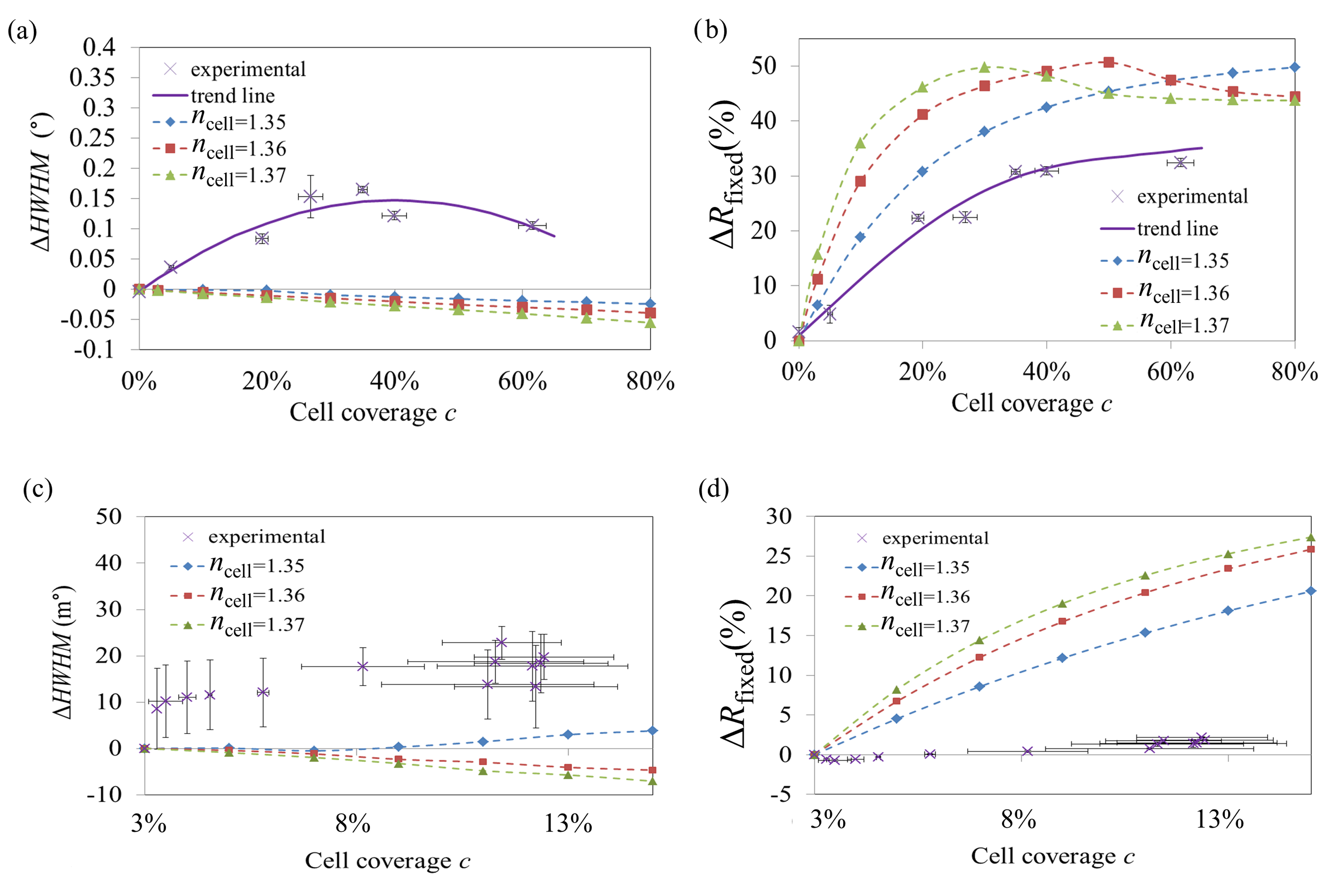

Supplement: Figure S9 — Comparison of experimental LRSPR HWHM and R fixed to those predicted by the effective-RI LRSPR model. The parameter R fixed represents the readable signal of typical biosensing experiments, it is obtained by the monitoring the intensity of the signal for a constant angle chosen to be close to, but smaller than, θ res, in the linear region of the SPR dip. Dependence of HWHM (a) and R fixed (b) with respect to the cell coverage. Dependence of HWHM (c) and R fixed (d) with respect to the spreading of cells. The simulations are plotted in dashed curves (n cell = 1.35: diamond, n cell = 1.36: squares, n cell = 1.37: triangles). The linear fitting in (a) yielded n cell = 1.360. Error bars represent the standard errors. (TIF) [file pone.0107978.s009.tif]
