# Supplementary material for: Systematic Study of the Surface Plasmon Resonance Signals Generated by Cells for Sensors with Different Characteristic Lengths
Source: PLoS One. 2014 Oct 23;9(10):e107978. doi: 10.1371/journal.pone.0107978 (PMC4207693; doi:10.1371/journal.pone.0107978)
Supplement: Appendix S1 — Relationship between the plasmonic waves and the cells. (DOC) [file pone.0107978.s010.doc]

#### S.1 Propagation lengths

The propagations lengths were computed numerically from dispersion equations using a program written in Maple. In case of solutions that were hardly converging due to the complexity of the dispersion equation, the function *RootFinding:-Analytic* from the *RootFinding* library was found to be the most efficient in yielding a solution. For accuracy the number of digit of float numbers was brought to 20. Indices of refraction and permittivity values were taken either from the literature or from previous measurements.

The propagation of surface plasmons (SP) is not infinite since plasmonic waves are bound at the interface of a dielectric medium and a lossy medium. The propagation length is here defined as the distance at which the electromagnetic field has dropped by a factor *e* in the direction of propagation. In order to calculate the propagation lengths, *L*_prop_, for the SPR structures utilized in this study, the complex propagation constant of the structure, *β* _SP_, was determined, whose imaginary part is related to the propagation length by:

$L_{\text{prop}}=\frac{1}{\mathrm{Im}\left(\beta_{SP}\right)}$, (S1)

Regarding the real part of  *β* _SP_, the SPR occurs when the light momentum matches that of the surface plasmons and one can write:

$n_{0}\cdot\sin\theta_{0}=\mathrm{Re}\left(\beta_{SP}\right)$, (S2)

where *n*_0_ and *θ*_0_ are the refractive indices and the incident angle of the glass medium. To calculate the *L*_prop_ of a conventional SPR (cSPR) structure, the propagation constant of an ideal two-semi-infinite layer structure, *β* _SP_^0^, needs to be completed with an additional term, Δ*β*, relating the losses induced by the presence of medium 0 (the prism in the present system) and the finiteness of the medium 1 (the gold layer). Following Raether, we can write for a cSPR structure :

*β* _SP_ = *β* _SP_^0^+ Δ*β*, (S3)

The permittivity constants of two-semi-infinite layers are assumed to be those of gold metal, *ε*_1_ and cover medium, *ε* _2_. It is well known that solving the Maxwell equations of this system yield the following dispersion equation:

, (S4)

The real part of *β* _SP_^0^ expresses momentum of the SPs, while the imaginary part relates the internal damping of the structure. Under the condition that

$e^{2\cdot i\cdot\kappa\cdot d}<<1$, (S5)

where *i* = (-1)^1/2^, *κ* is the propagation constant normal to the surface in the metal layer according to the relation *κ =* (*k*_0_^2^ *ε*_1_‑ *k*_0_^2^ *ε*_0_.sin^2^*θ* _0_)^1/2^ , with *k*_0_ being propagation constant of light in vacuum and *ε*_0_ the permittivity of the prism medium; and *d* the thickness of the metal layer, the additional term can be approximated to the following:

$\Delta\beta\approx2k_{0}\cdot r_{01}\cdot e^{2\cdot i\cdot\kappa\cdot d}\cdot\left(\frac{\varepsilon_{1}\cdot\varepsilon_{2}}{\varepsilon_{1}+\varepsilon_{2}}\right)^{\frac{3}{2}}\cdot\frac{1}{\varepsilon_{1}-\varepsilon_{2}}$, (S6)

where *r*_01_ is the p-polarized light Fresnel coefficient of reflection from medium 0 to medium 1. The real part of Δ*β* modifies slightly the resonance conditions, whereas the imaginary part accounts for the radiation losses. The aforementioned condition (S5) was found to be fulfilled with the parameters (*ε*_0_= 3.4100, *ε*_1_= ‑ 12.3 + 1.3*i*, *d*= 50 nm, *ε*_2_= 1.7913) as $\left|e^{2\cdot i\cdot\kappa\cdot d}\right|=0.023$ for a cSPR structure. Using equations (S1), (S3), (S4) and (S6), one can find *L*_prop_= 26.5 μm. This value is larger than that for two semi-infinite layers (equation (S4) alone), *L*_prop_= 7.8 μm, due to the fact that in the semi-infinite gold medium the internal losses are affecting the whole electromagnetic fields in the semi-space. It is worth noting that for simplicity, in the previous calculations the thin chromium layer was omitted.

In order to verify the calculated *L*_prop_ values, an alternative approach was used based on the dispersion equation of a three-layer structure:

$\left(\frac{\varepsilon_{0}}{\kappa_{0}}+\frac{\varepsilon_{1}}{\kappa_{1}}\right)\cdot\left(\frac{\varepsilon_{1}}{\kappa_{1}}+\frac{\varepsilon_{2}}{\kappa_{2}}\right)+\left(\frac{\varepsilon_{1}}{\kappa_{1}}-\frac{\varepsilon_{0}}{\kappa_{0}}\right)\cdot\left(\frac{\varepsilon_{2}}{\kappa_{2}}-\frac{\varepsilon_{1}}{\kappa_{1}}\right)\cdot e^{-2\cdot i\cdot\kappa_{1}\cdot d_{1}}=0$. (S7)

The three-layer structure was implemented in the Maple program and led to *L*_prop_= 35.8 μm showing a discrepancy with the Raether method, most likely due the approximations in expression (S6). Furthermore, if the four-layer structure dispersion equation (S8) (see below) was used for cSPR taking into account the thin chromium layer, *L*_prop_ is comprised between 29.3 μm and 24.6 μm for 1 nm and 2 nm of Cr respectively. *L*_prop_ is, in the case of an adhesive Cr layer, diminished compared to the case without due to the additional internal losses brought by the very absorbent chromium. Lastly with cSPR, the case of cell-covered surfaces was considered and if the cover refractive index is taken to be that of cells, regardless the approach used, *L*_prop_ is only reduced by less than 2 µm. The latter is based on the assumption that the effective refractive index of a cell monolayer is *n*_cell_= 1.35 ‑ 1.37.

In the case of the long-range SPR (LRSPR) structures, the condition (S5) is not fulfilled, mainly due to the very small thickness of the gold film ($\left|e^{2\cdot i\cdot\kappa\cdot d}\right|=0.24$) and therefore predicting the propagation length of LRSPR sensor in a similar fashion to cSPR structures would be very inaccurate. The only method available to do so involved using the dispersion equation of a four-layer structure, which was solved for *β*_SP_ :

$\begin{matrix} \left(\frac{\varepsilon_{3}}{\kappa_{3}}+\frac{\varepsilon_{2}}{\kappa_{2}}\right)\cdot\left(\frac{\varepsilon_{2}}{\kappa_{2}}+\frac{\varepsilon_{1}}{\kappa_{1}}\right)\cdot\left(\frac{\varepsilon_{1}}{\kappa_{1}}+\frac{\varepsilon_{0}}{\kappa_{0}}\right)+\left(\frac{\varepsilon_{3}}{\kappa_{3}}-\frac{\varepsilon_{2}}{\kappa_{2}}\right)\cdot\left(\frac{\varepsilon_{2}}{\kappa_{2}}-\frac{\varepsilon_{1}}{\kappa_{1}}\right)\cdot\left(\frac{\varepsilon_{1}}{\kappa_{1}}+\frac{\varepsilon_{0}}{\kappa_{0}}\right)\cdot e^{-2\cdot i\cdot\kappa_{2}\cdot d_{2}}+ \\ \left(\frac{\varepsilon_{3}}{\kappa_{3}}+\frac{\varepsilon_{2}}{\kappa_{2}}\right)\cdot\left(\frac{\varepsilon_{2}}{\kappa_{2}}-\frac{\varepsilon_{1}}{\kappa_{1}}\right)\cdot\left(\frac{\varepsilon_{1}}{\kappa_{1}}-\frac{\varepsilon_{0}}{\kappa_{0}}\right)\cdot e^{-2\cdot i\cdot\kappa_{1}\cdot d_{1}}+ \\ \left(\frac{\varepsilon_{3}}{\kappa_{3}}+\frac{\varepsilon_{2}}{\kappa_{2}}\right)\cdot\left(\frac{\varepsilon_{2}}{\kappa_{2}}+\frac{\varepsilon_{1}}{\kappa_{1}}\right)\cdot\left(\frac{\varepsilon_{1}}{\kappa_{1}}-\frac{\varepsilon_{0}}{\kappa_{0}}\right)\cdot e^{-2\cdot i\cdot\left(\kappa_{1}\cdot d_{1}-2\cdot i\cdot\kappa_{2}\cdot d_{2}\right)}=0 \end{matrix}$ (S8)

It was not possible to numerically solve equation (S8) for *β*_SP_ in the case of the four-layer structure with 20 nm and 800 nm of Cytop polymer due to algorithm divergence. However, as can be seen from Figure S4, in the case of 850 nm of Cytop polymer, the numerical calculations indicates that the propagation is greater than 180 μm. Note that for the cSPR structure *ε* _1_= ‑ 12.3 + 1.3*i* and for the LRSPR structure *ε* _2_= ‑ 10.751 + 1.3823*i* which is a correction arising from experimental measurements. If the refractive index of the cover is taken to be that of a cell layer on the sensor surface (*n*_cell_= 1.35, *n*_cell_= 1.36 and *n*_cell_= 1.37), the propagation length is diminished by 25 to 35 %, but still remains over 100 µm.

#### S.2 Penetration depths

In a stratified SPR structure, the electromagnetic (EM) fields are evanescent. The penetration depth, *L*_p_, also referred to as the probing distance, can be calculated as the distance, in the direction normal to the surface (*z* direction), at which the strength of the EM field has decayed by a factor *e*. In biosensing, the penetration depth of interest is the one from the metal/cover medium interface into the cover medium. Firstly in this paragraph, the penetration depth at resonance is of interest (i.e. *θ* = *θ*_res_). From the expression of an electromagnetic plane wave it is easily shown that the *L*_p_ is equal to the reciprocal of the imaginary part of the propagation constant, *κ*, normal to the surface of the cover medium, which, in turn, can be written:

(S9)

where *θ*_res_ is the angle of incidence at which resonance occurs, *ε* _s_ is the permittivity of the cover medium, i.e. *ε*_3_ in a four-layer structure. Equation (S9) gives *L*_p_= 180 nm and *L*_p_= 530 nm for typical cSPR and LRSPR (using Cytop as the buffer layer) structures, respectively, for aqueous-based media such as PBS or DMEM.

In order to evaluate the maximal deviations of *L*_p_, the extreme situation of monolayers of cells on the SPR structures was assessed. First, cells on cSPR sensors, with *n*_cell_= 1.35, *n*_cell_= 1.36 and *n*_cell_= 1.37, yielded *L*_p_= 168 nm, *L*_p_= 160 nm and *L*_p_= 153 nm, respectively. On the other hand, in the situation of monolayers of cells on LRSPR structures, with *n*_cell_= 1.35, *n*_cell_= 1.36 and *n*_cell_= 1.37, *L*_p_= 674 nm, *L*_p_ = 884 nm and *L*_p_= 1368 nm were respectively calculated. It can be noted that in the case of cSPR, since the SPR dip occurs for angles far from the critical angle, *L*_p_ does not vary much across the dip. It is reasonable to consider the previous *L*_p_ values as an mean value across the spectrum around resonance. However, for LRSPR, the proximity of the critical angle and the SPR dip requires *L*_p_ to be calculated for each value of the spectrum as it varies drastically. Hence, Eq. (S9) shall receive *θ* instead of *θ*_res_ as will be needed for the calculation of Eq. (4) and (6).

#### S.3 Conclusions the morphology and the plasmonic wave characteristic parameters

S.3.1 Propagation with respect to the lateral size of round cells

From the paragraph 2.5 it was found that round cells are about 16 µm in diameter. Although some discrepancies in the calculated *L*_prop_ for cSPR structures have been noticed depending on the methodology used, the different approaches agree to the extent that the *L*_prop_ is about 1‑2 times the lateral size of a typical round cell (i.e. in the absence of significant spreading). Due to the fact that the plasmonic waves have similar dimensions than the lateral size of the cells, it is not reasonable to approximate the cover medium as an effective or averaged medium with a unique refractive index function of the density of cells.

On the other hand, in the case of LRSP-supporting structures, *L*_prop_ is greater than 5 times the lateral size of a round cell. Hence it is not be not possible to treat the responses of individual cells separated as the plasmonic waves sense an effective medium formed of cells and cover solution. One could therefore envisage using the effective medium theory in order to determine the effective index of the cover medium composed of the cells and the cover solution. However, in this theory, the period of the oscillations needs to be much larger than the inhomogeneities, not the distances over which they propagate. In the considered case, the period of an oscillation of a plasmonic wave is *λ*/(*n*_0_.sin*θ*_0_) is between 440 nm and 470 nm and hence precludes the use of the effective medium theory with cell, the size of which being in the order of 20‑fold larger. In the main content of the article, an effective refractive index is calculated based on the methodology used for waveguides.

S.3.2 Penetration with respect to the height of round cells

Regarding the *z* direction, the height of round cells is much larger than the penetration depth. Furthermore, based on confocal fluorescence microscopy images regularly reported in the literature, it is assumed that the shape of a round cell on a surface is actually cylindrical with a circular base of diameter being 2x*R*_cell_ (i.e. 16.1 µm) and a height equalling 4*R*_cell_/3 (i.e. ~ 10.7 µm) . Since the penetration depth for cSPR structures is much smaller than the cell dimensions, a round cell can locally be considered as a semi-infinite medium in the *z* direction. However, a small part of the LRSPR dip close to the critical angle will be affected by the penetration depth being of the order of the cell average height, which will be included in the effective-refractive-index model.

S.3.3 Propagation with respect to the lateral size of spread cells

In the case of cSPR, the propagation length reaches approximately the typical lateral dimension of a spread cell. And similar conclusions as in S.3.1 can be drawn. The LRSPR propagation lengths are still much greater than the lateral dimension of spread cells and therefore even in the *cellular spreading* scheme, cell-free and cell-covered areas must not be considered independent.

S.3.4 Penetration with respect to the height of spreading cells

In the case of spread cells, the average thickness is 2.3 μm which is over 10-fold larger than the cSPR penetration depth in DMEM. The spreading cells can therefore still be considered as a semi-infinite medium and *R*_cell_ in Eq. (1) shall remain the same irrespective to cell average height. In the case of LRSPR sensor, the evanescent fields are not completely attenuated within the cells and a substantial proportion still remains beyond the cell average height. Therefore the cells cannot be considered as a semi-infinite medium and the average height needs to be taken into account in the model described by Eq. (4) and (6).

1. Raether H (1988) Surface plasmons on smooth surfaces. Surface Plasmons on Smooth and Rough Surfaces and on Gratings: Springer Berlin Heidelberg. pp. 4-39.

2. C.A. Ward, K. Bhasin, R. J. Bell, R. W. Alexander, Tyler I (1975) Multimedia dispersion relation for surface electromagnetic waves. The journal of chemical Physics 62: 3.

3. Cortès S, Villiers CL, Colpo P, Couderc R, Brakha C, et al. (2011) Biosensor for direct cell detection, quantification and analysis. Biosensors and Bioelectronics 26: 4162-4168.
